# Differential Ca^2+^ handling by isolated synaptic and non-synaptic mitochondria: roles of Ca^2+^ buffering and efflux

**DOI:** 10.3389/fnsyn.2025.1562065

**Published:** 2025-05-27

**Authors:** Jyotsna Mishra, Kyle Bevers, Keguo Li, Armaan Zare, James S. Heisner, Ailing Tong, Wai-Meng Kwok, David F. Stowe, Amadou K. S. Camara

**Affiliations:** ^1^Department of Anesthesiology, Medical College of Wisconsin, Milwaukee, WI, United States; ^2^Department of Pharmacology and Toxicology, Medical College of Wisconsin, Milwaukee, WI, United States; ^3^Cardiovascular Center, Medical College of Wisconsin, Milwaukee, WI, United States; ^4^Cancer Center, Medical College of Wisconsin, Milwaukee, WI, United States; ^5^Department of Physiology, Medical College of Wisconsin, Milwaukee, WI, United States; ^6^Department of Biomedical Engineering, Medical College of Wisconsin and Marquette University, Milwaukee, WI, United States

**Keywords:** synaptic mitochondria, non-synaptic mitochondria, Ca^2+^ buffering, Ca^2+^ efflux, bioenergetics

## Abstract

Mitochondria regulate intracellular calcium ion (Ca^2+^) signaling by a fine-tuned process of mitochondrial matrix (m) Ca^2+^ influx, mCa^2+^ buffering (sequestration) and mCa^2+^ release (Ca^2+^ efflux). This process is critically important in the neurosynaptic terminal, where there is a simultaneous high demand for ATP utilization, cytosolic (c) Ca^2+^ regulation, and maintenance of ionic gradients across the cell membrane. Brain synaptic and non-synaptic mitochondria display marked differences in Ca^2+^ retention capacity. We hypothesized that mitochondrial Ca^2+^ handling in these two mitochondrial populations is determined by the net effects of Ca^2+^ uptake, buffering or efflux with increasing CaCl_2_ boluses. We found first that synaptic mitochondria have a more coupled respiration than non-synaptic mitochondria; this may correlate with the higher local energy demand in synapses to support neurotransmission. When both mitochondrial fractions were exposed to increasing mCa^2+^ loads we observed decreased mCa^2+^ sequestration in synaptic mitochondria as assessed by a significant increase in the steady-state free extra matrix Ca^2+^ (ss[Ca^2+^]_e_) compared to non-synaptic mitochondria. Since, non-synaptic mitochondria displayed a significantly reduced ss[Ca^2+^]_e_, this suggested a larger mCa^2+^ buffering capacity to maintain [Ca^2+^]_m_ with increasing mCa^2+^ loads. There were no differences in the magnitude of the transient depolarizations and repolarizations of the membrane potential (ΔΨ_m_) and both fractions exhibited similar gradual depolarization of the baseline ΔΨ_m_ during additional CaCl_2_ boluses. Adding the mitochondrial Na^+^/Ca^2+^ exchanger (mNCE) inhibitor CGP37157 to the mitochondrial suspensions unmasked the mCa^2+^ sequestration and concomitantly lowered ss[Ca^2+^]_e_ in synaptic *vs*. non-synaptic mitochondria. Adding complex V inhibitor oligomycin plus ADP (OMN + ADP) bolstered the matrix Ca^2+^ buffering capacity in synaptic mitochondria, as did Cyclosporin A (CsA), in non-synaptic. Our results display distinct differences in regulation of the free [Ca^2+^]_m_ to prevent collapse of ΔΨ_m_ during mCa^2+^ overload in the two populations of mitochondria. Synaptic mitochondria appear to rely mainly on mCa^2+^ efflux via mNCE, while non-synaptic mitochondria rely mainly on P_i_-dependent mCa^2+^ sequestration. The functional implications of differential mCa^2+^ handling at neuronal synapses may be adaptations to cope with the higher metabolic activity and larger mCa^2+^ transients at synaptosomes, reflecting a distinct role they play in brain function.

## 1 Introduction

Brain mitochondria (m) orchestrate diverse functions including energy transduction (oxidative phosphorylation), neuronal excitability and synaptic neurotransmission and its regulation. A key component in these processes is the interplay of cytosolic (c) and mCa^2+^ dynamics. Mitochondria contribute to the shaping of cCa^2+^ transients by regulating mCa^2+^ uptake, buffering and release. In this process, mitochondria regulate energy metabolism, synaptic activity, vesicular exocytosis, gene expression, fission/fusion, and mitophagy (Pivovarova and Andrews, 2010; Jung et al., 2020). In metabolically active tissues like the brain, mitochondria take up Ca^2+^ mainly via the outer mitochondrial membrane (OMM) voltage dependent anion channel 1 (VDAC1) into the intermembrane space (IMS) (Camara et al., 2010; Camara et al., 2011; Camara et al., 2017). From the IMS, Ca^2+^ enters the inner mitochondrial membrane (IMM) via the mCa^2+^ uniporter (mCU) into the matrix (Camara et al., 2010; Baughman et al., 2011; De Stefani et al., 2011; Camara et al., 2017; Mishra et al., 2017). The large negative electrochemical gradient across the IMM (ΔΨ_m_: −180 to −200 mV) is the main driving force for mCU-mediated Ca^2+^ uptake.

Mitochondrial Ca^2+^ release is performed primarily by the mitochondrial Na^+^/Ca^2+^/Li^+^ exchanger (mNCLX) (Palty et al., 2010), a.k.a. mitochondrial Na^+^/Ca^2+^ exchanger (mNCE). Another plausible mechanism for mCa^2+^ release is via the putative Na^+^-independent Ca^2+^/H^+^ exchanger (mCHE) as examined in cardiac myocytes (Haumann et al., 2018; Natarajan et al., 2020; Haumann et al., 2010) even though mCHE is reported to be more active in non-excitable cells (Rottenberg and Marbach, 1990), like hepatocytes. In the matrix, excess free Ca^2+^ is buffered primarily by inorganic phosphate (P_i_), but also by adenine nucleotides (Haumann et al., 2010; Haumann et al., 2018; Mishra et al., 2019) and matrix proteins (Chalmers and Nicholls, 2003; Starkov, 2010; Blomeyer et al., 2013). The buffering system allows mitochondria to accumulate large quantities by mCa^2+^ uptake, while maintaining free [Ca^2+^]_m_ in the physiological range (~100 nM) (Chalmers and Nicholls, 2003). Thus, free [Ca^2+^]_m_ is managed by a balance in mCa^2+^ influx, mCa^2+^ buffering, and mCa^2+^ efflux. Hence, impaired cCa^2+^ cycling can lead to disruption of [Ca^2+^]_m_ homeostasis, leading to mCa^2+^ overload, matrix swelling, collapse of the membrane potential, ΔΨ_m_, and possibly to opening of the mitochondrial permeability transition pore (mPTP), with IMM rupture and total Ca^2+^ release. This Ca^2+^ dysregulation in synaptic terminals is implicated in several neurodegenerative diseases (Du et al., 2008; Kalani et al., 2018).

**Figure 1:**
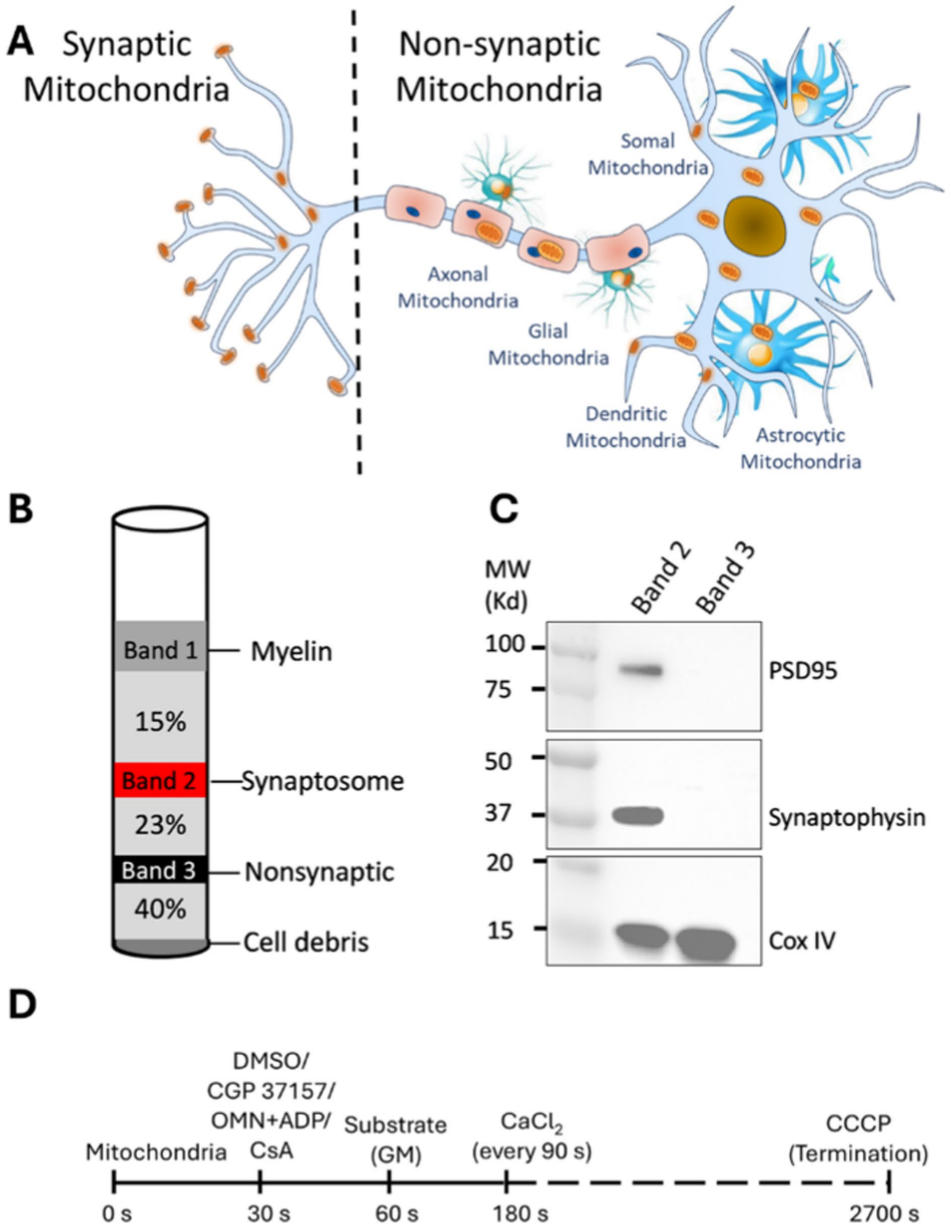
Synaptic and non-synaptic mitochondria isolation and experimental timeline to study Ca^2+^ handling and bioenergetics in isolated synaptic and non-synaptic mitochondria. **(A)** Compartmental distribution of mitochondria in subcellular location of neurons and glial cells. Synaptic mitochondria are located within the synaptosome while non-synaptic mitochondria are derived from soma, glial and vascular cells. **(B)** Schematic diagram of visible bands seen upon ultracentrifugation as described in materials and methods. The synaptosome (band 2; red color) and non-synaptic (band 3; black color) fractions are indicated in their respective gradients following ultracentrifugation. **(C)** Representative western blot of synaptosome markers in isolated synaptic (band 2) and non-synaptic (band 3) fractions. **(D)** Timeline of the experimental protocol (in seconds) for isolated synaptic and non-synaptic mitochondria. Different interventions, such as CGP 37157 (2 μM), oligomycin (OMN; 10 μM) + ADP (250 μM) and CsA (0.5 μM) were added at 30 s, followed by the addition of the substrates [Na^+^-glutamate + Na^+^-malate (GM)] at 60 s. At 180 s, 40 μM of CaCl_2_ was added, followed by sequential additions of 40 μM CaCl_2_ at every 90 s intervals. CCCP (10 μM) was added at the end of each experiment to achieve the maximal dye release from mitochondria to terminate the experiment.

The brain consists of a highly heterogeneous ensemble of cells (e.g., neurons, glia, including astrocytes, and endothelial cells) with distinct anatomical and functional roles. Mitochondrial function, content and morphology vary among cell types, and within cells to account for their different physiological roles in the entire brain (Davis and McLean, 1987; Fecher et al., 2019; Pekkurnaz and Wang, 2022). Mitochondria are abundant in neurons, especially at the synapses (Vos et al., 2010), whereas fewer mitochondria per cell volume are found in glial and endothelial cells (Kannurpatti, 2017) (Figure 1). The high density of mitochondria in synapses are needed to meet the enhanced production of ATP, via oxidative phosphorylation, that is necessary for the high-energy demand to maintain, for example, ionic homeostasis via the Na^+^/K^+^ and Ca^2+^-ATPase pumps during synaptic neurotransmission (Hollenbeck, 2005; Magistretti and Allaman, 2015; Bordone et al., 2019). These processes are necessary for neurons to maintain cellular ionic conditions, i.e., Na^+^ and K^+^, and Ca^2+^ gradients and the cell membrane potential (Meir et al., 1999; Billups and Forsythe, 2002; Verma et al., 2022) to maintain the capability for neurotransmission.

The heterogeneity of the brain mitochondrial population is notable at the neuronal level (Pekkurnaz and Wang, 2022). Synaptic mitochondria are located mainly in the synaptosome, while non-synaptic mitochondria are derived primarily from neuronal soma and axons, and non-neuronal (glia, and endothelial) cells of the brain (Brown et al., 2006; Kulbe et al., 2017; Hill et al., 2020; Olesen et al., 2020). In a previous study, Naga et al. (2007) showed that synaptic mitochondria isolated from rat cerebral cortex are more sensitive to Ca^2+^ overload than are non-synaptic mitochondria and have higher synaptic mitochondrial levels of cyclophilin-D (Cyp D), a matrix protein implicated in the regulation of the mPTP opening (Naga et al., 2007; Du et al., 2008). In addition, synaptic mitochondria display higher vulnerability to oxidative damage (Du et al., 2010) and exhibit a higher mCa^2+^ level (Brown et al., 2006; Kulbe et al., 2017) than non-synaptic mitochondria during aging and in neurological disorders (Reddy and Beal, 2008).

These unique features in synaptic mitochondria are likely a result of the high cCa^2+^ flux demands for instant ATP resupply during neurotransmission (Datta and Jaiswal, 2021). Indeed, studies have demonstrated differential mCa^2+^ handling in mitochondria isolated from synaptic and non-synaptic brain tissue. As noted above, synaptic mitochondria retained less Ca^2+^ during CaCl_2_ pulse challenges and had more Cyp D than non-synaptic mitochondria (Brown et al., 2006; Naga et al., 2007). However, knowledge of the detailed kinetics of synaptic mCa^2+^ dynamics, particularly mCa^2+^ uptake, buffering and efflux, and its implications for matrix Ca^2+^ handling is limited.

In this study, we sought to characterize the dynamics of mCa^2+^ handling in synaptic and non-synaptic mitochondria isolated from rat cerebral cortex and to assess the physiological and pathophysiological implications of their differential modes of mCa^2+^ handling. To characterize mCa^2+^ handling in synaptic and non-synaptic mitochondria we investigated the properties of: (1) mCa^2+^ extrusion via the mNCE using its inhibitor CGP 37157 (CGP), (2) mCa^2+^ buffering by changing the matrix ADP, ATP pool using a combination of oligomycin and ADP (OMN + ADP), and (3) mCa^2+^ buffering by cyclosporin A (CsA), a Cyp D inhibitor (Camara et al., 2010), but which, via a P_i_-dependent (Mishra et al., 2019) Ca^2+^ buffering mechanism, also enhances mCa^2+^ sequestration during CaCl_2_ pulse challenges in cardiomyocytes (Haumann et al., 2018; Mishra et al., 2019). Based on these observations, we show that the differences in mCa^2+^ handling between synaptic and non-synaptic mitochondria is attributed in large part to their differences in mCa^2+^ efflux and mCa^2+^ buffering mechanisms.

## 2 Materials and methods

### 2.1 Materials

All chemical reagents were purchased from Sigma-Aldrich (St. Louis, MO, United States), unless otherwise stated. CGP37157 (CGP) and Cyclosporin A (CsA) were purchased from Tocris Bioscience (Bristol, United Kingdom). Fluorescent probes Fura-4F penta-K^+^ salt and tetramethyl-rhodamine methyl ester perchlorate (TMRM) were purchased from Life Technologies (Eugene, OR).

### 2.2 Animal care

Male Sprague Dawley (SD) rats weighing 300–400 grams were procured from Envigo. Rats were 8 to 12 weeks old at the time of experimentation. All procedures were carried out in accordance with the National Institutes of Health (NIH) Guide for the Care and Use of Laboratory Animals (NIH Publication No. 85-23, revised 1996) and were approved by the Institutional Animal Care and Use Committee (IACUC) of the Medical College of Wisconsin.

### 2.3 Synaptic and non-synaptic mitochondria isolation

Synaptic and non-synaptic mitochondria were isolated from SD rats according to the procedure described by Naga et al. (2007), with some modifications. Briefly, the rats were anesthetized with a combination of an intraperitoneal injection of inactin (0.05 mg) for sedation and heparin (500 units) for anticoagulation. After total sedation and decapitation, the brain was rapidly harvested and the cerebral cortex was separated and minced into ice-cold Na^+^-free isolation buffer containing in mM: 200 mannitol, 50 sucrose, 5 KH_2_PO_4_, 5 MOPS, 1 EGTA, and 0.1% bovine serum albumin at pH 7.15 (adjusted with KOH). The minced tissue was suspended in a 5 mL ice-cold isolation buffer containing 0.05% nagarse, a protease, and then homogenized using a Dounce homogenizer. The homogenate was centrifuged at 1,300 g for 5 min. The supernatant was layered on a discontinuous Percoll gradient (2.5 mL each of 15, 23, 40% by volume Percoll in isolation buffer) and centrifuged at 34,000 g for 9 min with a Beckman Optima XPN ultracentrifuge. Band 2 (between 15 and 23% Percoll) and Band 3 (between 23 and 40% Percoll) were carefully removed using a 25-gauge Hamilton syringe; these constitute, respectively, the synaptic and non-synaptic mitochondrial fractions (Figure 1).

Each fraction was resuspended in 5 mL total volume of isolation buffer. A 50 μL aliquot of 2% digitonin was added to both fractions and allowed to incubate on ice for 10 min. Both suspensions were centrifuged at 16,500 g for 15 min and the supernatants were discarded. After resuspension of the pellets in separate aliquots of 5 mL isolation buffer, both fractions were re-centrifuged at 8,000 g for 10 min and the supernatants discarded. Protein concentration was determined by the Bradford method and the final mitochondrial suspensions were adjusted to 6.25 mg protein/mL in the isolation buffer. All centrifugations were performed at 4°C, and all buffers used for the isolation were kept on ice during the procedure. The isolated mitochondria were kept on ice for the duration of the experiments. All experiments on mitochondria function were conducted at room temperature (~25°C), as we have reported previously (Blomeyer et al., 2013; Blomeyer et al., 2016; Haumann et al., 2018; Mishra et al., 2019; Mishra and Camara, 2022; Sun et al., 2022).

### 2.4 Measurement of mitochondrial O_2_ consumption rate in synaptic and non-synaptic mitochondria

Respiratory Control Index (RCI) is a method to determine the functional integrity of mitochondria as described previously (Haumann et al., 2010; Mishra et al., 2019). Briefly, mitochondria were suspended in experimental buffer (see 2.5 and 2.6 below) and O_2_ consumption was measured, as described before (Riess et al., 2004; Riess et al., 2008; Yang et al., 2019; Gerdes et al., 2020; Sun et al., 2022; Kim et al., 2023), using the Clark-type electrode (model 1302, Strathkelvin Instruments) in a water jacketed and air-tight 500 μL chamber (Model MT200A). The mitochondrial suspension in the respiratory chamber was energized with complex I substrate, Na^+^-glutamate (0.5 mM) and Na^+^-malate (0.5 mM) (Na^+^–GM), to determine state 2 respiration, followed by the addition of ADP (250 μM), which results in a faster rate of O_2_ consumption, i.e., state 3 respiration. The conversion of all the added ADP to ATP, and the concomitant decrease in the O_2_ consumption rate represents state 4 respiration. The RCI, which determines the magnitude of coupling of oxidative phosphorylation, was defined as the ratio of state 3 to state 4 respiration. The lower RCI (see results) in non-synaptic mitochondria likely represents a degree of uncoupled respiration due to proton leak through the IMM. This process of H^+^ uptake is independent of H^+^ influx via the ATP synthase (complex V).

### 2.5 Measurement of mitochondrial Ca^2+^ handling in synaptic and non-synaptic mitochondria

Mitochondrial Ca^2+^ handling (i.e., uptake, sequestration and release), and mitochondrial membrane potential (ΔΨ_m_) experiments were monitored over time using a Photon Technology Instrument fluorescence spectrophotometer (PTI; Qm-8 Horiba, Birmingham NJ, United States) (Haumann et al., 2010; Mishra et al., 2019). The experiments started with 0.5 mg isolated mitochondria added in 1 mL Na^+^-free experimental buffer containing in mM: 130 KCl, 5 K_2_ HPO_4_, 20 MOPS, 0.1% BSA and 40 μM EGTA at pH 7.15 (adjusted with KOH) along with 1 μM of the fluorescent dye Fura-4F K^+^ salt to measure extra-matrix Ca^2+^ ([Ca^2+^]_e_) transients (Figure 1D). At *t* = 30 s, vehicle or any drug being tested was added: these were CGP (2 μM) or OMN (10 μM) + ADP (250 μM), OMN alone, ADP alone, or CsA (0.5 μM). At *t* = 60 s, Na^+^–GM was added to energize mitochondria and to allow Na^+^ exchange with Ca^2+^ when CaCl_2_ is added. At *t* = 180 s and for every 90 s after that, 40 μM CaCl_2_ was added to the mitochondrial suspension in a cuvette placed in the PTI with continuous stirring in the PTI cuvette. At the end of each experiment, when mitochondria could not take up any additional CaCl_2_, the uncoupler CCCP (10 μM) was added to the suspension to cause maximal release of mCa^2+^. Changes in [Ca^2+^]_e_ were monitored at dual-excitation wavelengths *λ*_ex_ at 340/380 nm and a single emission wavelength *λ*_em_ at 510 nm.

### 2.6 Measurement of synaptic and non-synaptic mitochondrial membrane potential (ΔΨ_m_)

As in the CaCl_2_ pulse experiments above, each experiment started with 0.5 mg isolated mitochondria in 1 mL experimental buffer containing in mM: 130 KCl, 5 K_2_ HPO_4_, 20 MOPS, 0.1% BSA and 40 μM EGTA, and at pH 7.15 (adjusted with KOH) along with 1 μM TMRM, a permeant membrane potential (ΔΨ_m_) fluorescence dye. At *t* = 30 s, any drug being used was added: vehicle, CGP or OMN + ADP, OMN (alone), ADP (alone) or CsA. At *t* = 60 s, Na^+^–GM (0.5 M) was added; at *t* = 180 s and every 90 s after that, 40 μM CaCl_2_ was added. The TMRM fluorescence changes were measured at two excitations, *λ*_ex_ 546 and 573 nm, and a single emission *λ*_em_ 590 nm. CCCP was added as in section 2.6 to induce maximal depolarization of ΔΨ_m_.

### 2.7 Western blot assay of mitochondrial proteins involved in mCa^2+^ handling in synaptic and non-synaptic mitochondria

Purified synaptic and non-synaptic mitochondrial fractions were lysed in ice-cold RIPA lysis buffer (Thermo Fisher Scientific) supplemented with 1 mM phenylmethanesulfonyl fluoride (PMSF) and 1% protease inhibitor cocktail (Sigma-Aldrich). Lysates were vortexed and incubated for 30 min on ice, followed by centrifugation at 17,000 g for 15 min at 4°C. The supernatant was collected and quantified using the bicinchoninic acid assay (Thermo Fisher Scientific). The protein samples were reduced in 4× Laemmli sample buffer (Bio-Rad) containing 10% β-mercaptoethanol and denatured at 95°C for 5 min. Denatured proteins were resolved on 4–20% SDS-PAGE gels (Bio-Rad) and transferred onto nitrocellulose membranes (0.45 μm, Bio-Rad). The membranes were blotted with the following primary antibodies: mCU, Synaptophysin, PSD95, VDAC, ANT1, (Cell Signaling Technology) and Cyp D (abcam). The IMM protein, COX IV (Cell Signaling Technology), was used as the loading control. Of note, we did not report here the expression levels of mNCE, due to questionable reliability of the commercially available antibodies. The LI-COR infrared fluorescent mouse (925–68,020) and rabbit (925–32,211) secondary antibodies were used for visualization using a LI-COR Odyssey scanner at 680 and 800 nm, respectively. Band quantification (densitometry) was performed using ImageJ software.

### 2.8 Statistical analyses

All data were imported into a Microsoft Excel 2016 program for analysis. Any statistical difference in RCI was assessed using a paired student’s *t*-test. The student’s *t*-test for other experiments was paired when appropriate, i.e., synaptic vs. non-synaptic, synaptic with CGP vs. non-synaptic with CGP, synaptic with OMN + ADP vs. non-synaptic with OMN + ADP, and synaptic with CsA vs. non-synaptic with CsA, but was otherwise performed as a two-sample unequal variance test. All *t*-tests reported are two-tailed. To assess significance in the Ca^2+^ handling data (steady state extra-matrix Ca^2+^ levels), a student’s *t*-test was performed using the 5 s average of each experiment as a data point just prior to addition of each new CaCl_2_ bolus. Data are presented as means ± SEM.

## 3 Results

### 3.1 Isolation and validation of synaptic and non-synaptic mitochondrial population

Synaptic and non-synaptic mitochondrial fractions were isolated from freshly harvested brain tissue following a well-described protocol (Naga et al., 2007), with some modifications (Figure 1). Western blot analysis showed that the synaptic fractions are highly enriched with well-established protein markers including, postsynaptic density protein 95 (PSD95) and synaptophysin, (Glantz et al., 2007) and the IMM protein COXIV. The non-synaptic fraction showed a COXIV band, but no PSD95 and synaptophysin bands (Figure 1C). These results demonstrate an enriched abundant synaptosomal fraction in the synaptic population and validates our procedure to isolate synaptic and non-synaptic mitochondria from cerebral tissue as also described by others (Villasana et al., 2006; Kamat et al., 2014).

### 3.2 Differences in oxidative phosphorylation capacity of synaptic and non-synaptic mitochondria

**Figure 2:**
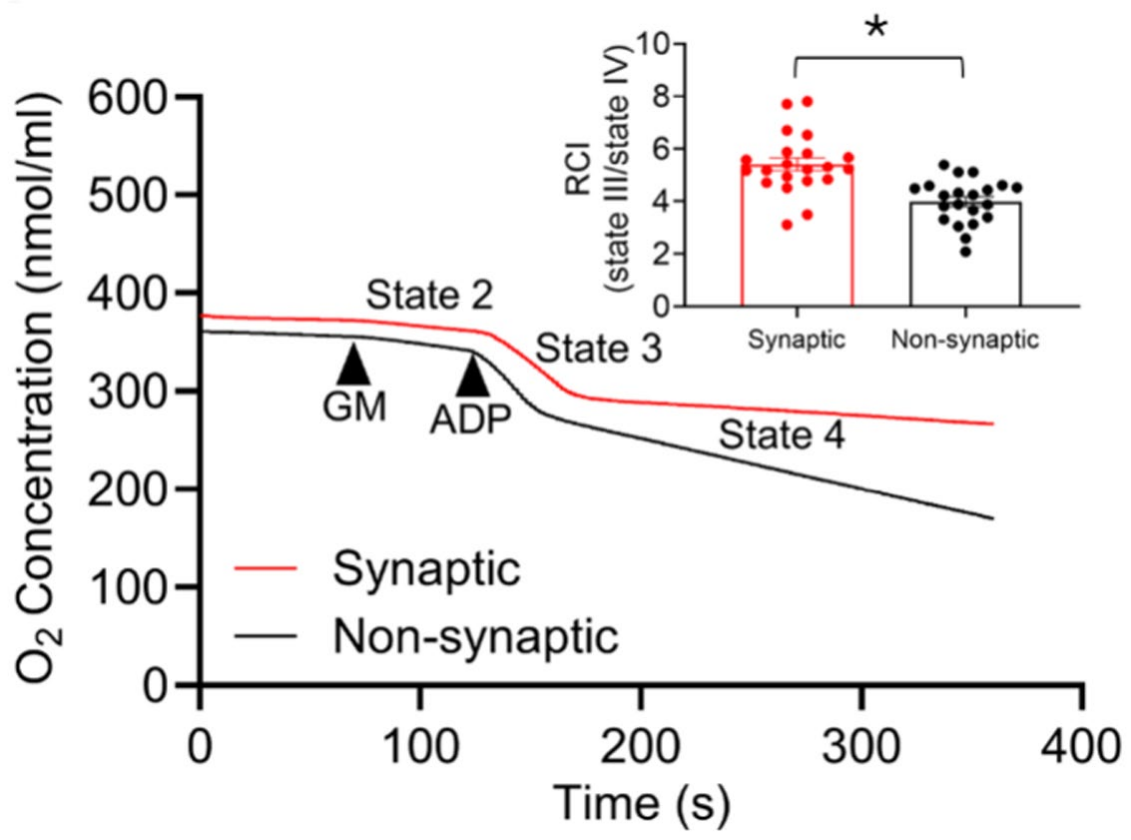
Representative traces of O_2_ consumption rate (OCR) of isolated synaptic and non-synaptic mitochondria. Complex I substrates [Na^+^-glutamate + Na^+^-malate (GM)] and ADP were sequentially injected to assess mitochondrial OCR, the slope, at the different respiratory states. The Respiratory Control Index (RCI) is derived from the ratio of the OCR during state 3 (in the presence of ADP) to the OCR during state 4 (after exhausting the added ADP) respiration. The insets show average RCI of synaptic and non-synaptic mitochondria. Data are expressed as mean ± SEM (^*^p < 0.0001).

To assess the capacity of oxidative phosphorylation in the two mitochondrial populations we assessed O_2_ consumption using the Clark-type electrode, as reported previously (Mishra et al., 2019; Mishra and Camara, 2022; Sun et al., 2022). Specifically, we measured the ADP-mediated increase in mitochondrial respiration after energizing with complex I substrate Na^+^-GM. We quantified the Respiratory Control Indexes, RCIs, i.e., the ratios of state 3 to state 4 respiration. Representative traces of mitochondrial respiration and a summary of RCIs (inset) are displayed in Figure 2. Synaptic and non-synaptic mitochondria exhibited similar state 2 and state 3 respirations (Supplementary Figure 1) but the mean RCI was significantly higher in synaptic mitochondria, 5.92 ± 0.43 vs. non-synaptic mitochondria, 4.17 ± 0.17 (Figure 2; inset). This is because state 4 respiration was faster in non-synaptic mitochondria than in synaptic mitochondria (Supplementary Figure 1), which indicates a degree of uncoupled respiration in non-synaptic mitochondria that is likely due to a mild H^+^ leak (H^+^ ions entering the matrix independent of the ATP-synthase).

### 3.3 Differences in [Ca^2+^]_m_ handling in synaptic and non-synaptic mitochondria

**Figure 3:**
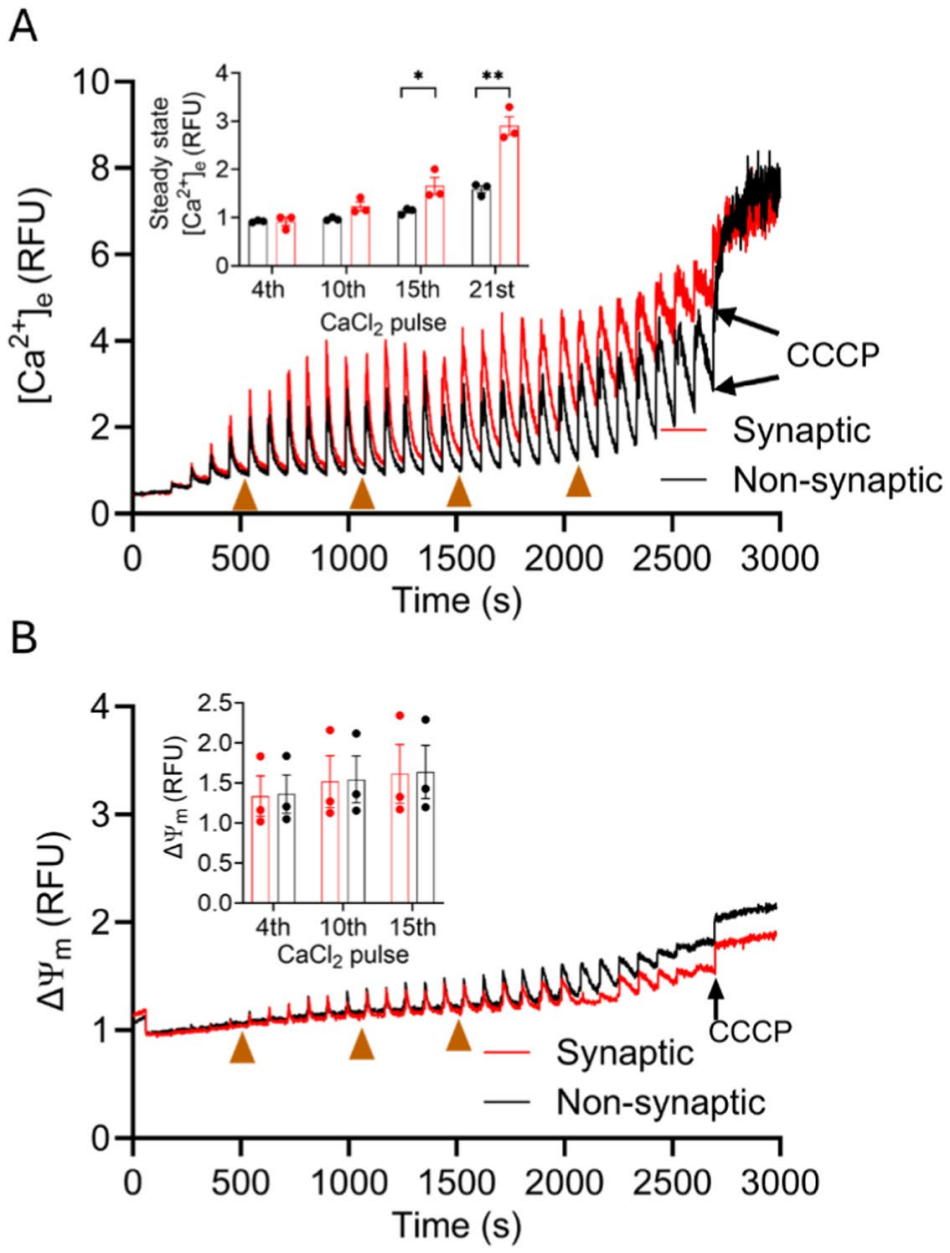
Comparison of synaptic and non-synaptic mitochondria Ca^2+^ handling. **(A)** Representative traces of extra-matrix Ca^2+^ ([Ca^2+^]_e_) measured with the Ca^2+^-sensitive ratiometric dye Fura-4FF in synaptic and non-synaptic mitochondria. **(B)** Change of ΔΨ_m_ in synaptic and non-synaptic mitochondria measured using the ΔΨ_m_ sensitive dye TMRM (tetramethylrhodamine methyl ester perchlorate). Synaptic (red trace) and non-synaptic (black trace) mitochondria were energized with complex I substrates, [Na^+^-glutamate + Na^+^-malate (GM)] at 60 s and 40 μM CaCl_2_ pulses were added at every 90 s, followed by addition of 10 μM CCCP at the end of the experiment. The insets show quantification of steady-state [Ca^2+^]_e_ **(A)** and ΔΨ_m_ **(B)**. Error bars represent mean ± SEM (^*^p < 0.05).

To comprehensively address the [Ca^2+^]_m_ handling in synaptic and non-synaptic mitochondria, we measured extra-mitochondrial Ca^2+^ ([Ca^2+^]_e_) (expressed as relative fluorescence units, RFU) during repetitive additions of 40 μM CaCl_2_ boluses, in the presence of 80 μM EGTA, at 90 s intervals until mitochondria stopped taking up the added exogenous CaCl_2_ (Figure 3). As shown in representative traces, each CaCl_2_ pulse is marked by a transient peak in the extra-matrix fluorescent signal for Ca^2+^ followed by a downward slope reaching a baseline steady-state level. For the 90 s inter-pulse interval, the steady-state (ss) [Ca^2+^]_e_ is defined as the net basal level of the extra-matrix free Ca^2+^ ([Ca^2+^]_e_) following mCa^2+^ uptake, sequestration, and efflux after each CaCl_2_ bolus (Blomeyer et al., 2016; Mishra et al., 2019). In this case, Figure 3 shows that non-synaptic mitochondria exhibited a more robust and sustained capacity for mCa^2+^ uptake and sequestration than the synaptic mitochondria.

To better elucidate the differences, we quantified ss[Ca^2+^]_e_ and plotted it against the cumulative additions of CaCl_2_, i.e., 160, 400, 600, and 840 μM. This corresponds to ss[Ca^2+^]_e_ at the 4th, 10th, 15th, and 21st CaCl_2_ pulse, respectively, and are shown as brown arrows in Figure 3. Synaptic mitochondria showed a gradual increase in ss[Ca^2+^]_e_, i.e., lesser retained extra-matrix Ca^2+^ that gradually accumulated in the experimental buffer. We reasoned this could be due to either a lesser mCa^2+^ uptake or to a greater mCa^2+^ release over time as additional boluses of CaCl_2_ were given. As presented below on the impact of the CaCl_2_ pulses on ΔΨ_m_, lesser Ca^2+^ uptake is not likely a contributing factor because mCa^2+^ uptake is dependent on a high ΔΨ_m_. In contrast, in non-synaptic mitochondria, CaCl_2_ pulses resulted in no change in ss[Ca^2+^]_e_ until the 21st pulse (Figure 3); this indicated that more mCa^2+^ was sequestered in mitochondria and so less mCa^2+^ was released. To assess the potential for differences in Ca^2+^ uptake rates in the two mitochondrial populations, mCU activity was quantified using a single exponential decay for each CaCl_2_ pulse to derive the decay constants. There was no significant difference in the Ca^2+^ decay constants between synaptic and non-synaptic mitochondria (Supplementary Figure 2). These results suggest a differential balance in the mechanism for handling mCa^2+^ in the two populations, with a likelihood for greater mCa^2+^ buffering in regulating [Ca^2+^]_m_ in non-synaptic mitochondria, and the possibility that mCa^2+^ efflux via mNCE as a potential mechanism for regulating mCa^2+^ in synaptic mitochondria, as presented later.

A high mitochondrial membrane potential (ΔΨ_m_) is the major determinant for mCa^2+^ uptake. When ΔΨ_m_ is fully charged, it leads to a rapid uptake of mCa^2+^ via the mCU. Excess free Ca^2+^ in the matrix causes depolarization and loss of ΔΨ_m_, which diminishes the drive for mCa^2+^ uptake. Therefore, we next investigated whether the differences in Ca^2+^ handling between synaptic and non-synaptic mitochondria could be attributed to differences in ΔΨ_m_ during CaCl_2_ pulse challenges. We examined changes in ΔΨ_m_ using the similar protocol (Figure 1D) as for measuring [Ca^2+^]_e_ with CaCl_2_ boluses. Figure 3B shows small transient depolarizations and repolarizations of ΔΨ_m_ with each bolus of CaCl_2_ in both synaptic and non-synaptic mitochondria. Basal ΔΨ_m_ gradually depolarized to similar levels in both populations; this suggest that the driving force for mCa^2+^ uptake was similar between the two groups. The Ca^2+^ transients also demonstrate that the differences in Ca^2+^ flux dynamics in the two mitochondrial populations are not related to differences in ΔΨ_m_ in response to repeated mCa^2+^ loading. In both populations during the latter CaCl_2_ pulse challenges, mitochondria stopped taking up additional Ca^2+^ because significant depolarization of ΔΨ_m_ terminated the driving force for mCa^2+^ uptake. Adding the protonophore CCCP after the end of the CaCl_2_ boluses validates the similar maximal depolarizations with complete collapse of ΔΨ_m_ in both mitochondrial fractions.

We have shown that in synaptic mitochondria, each CaCl_2_ pulse produced a new steady state (ss[Ca^2+^]_e_) slightly above the previous one. It is possible that this is due less to reliance on the mCa^2+^ sequestration system and more to an increase in efflux of mCa^2+^ via the mNCE. Unlike synaptic mitochondria, non-synaptic mitochondria maintained ss[Ca^2+^]_e_ at almost the basal level for an extended time during the CaCl_2_ boluses. We suspected that this is due to a greater capacity for mCa^2+^ buffering in non-synaptic mitochondria with a minor, or no effect, on mCa^2+^ efflux via mNCE.

### 3.4 Synaptic and non-synaptic mitochondria exhibit differences in mNCE function

**Figure 4:**
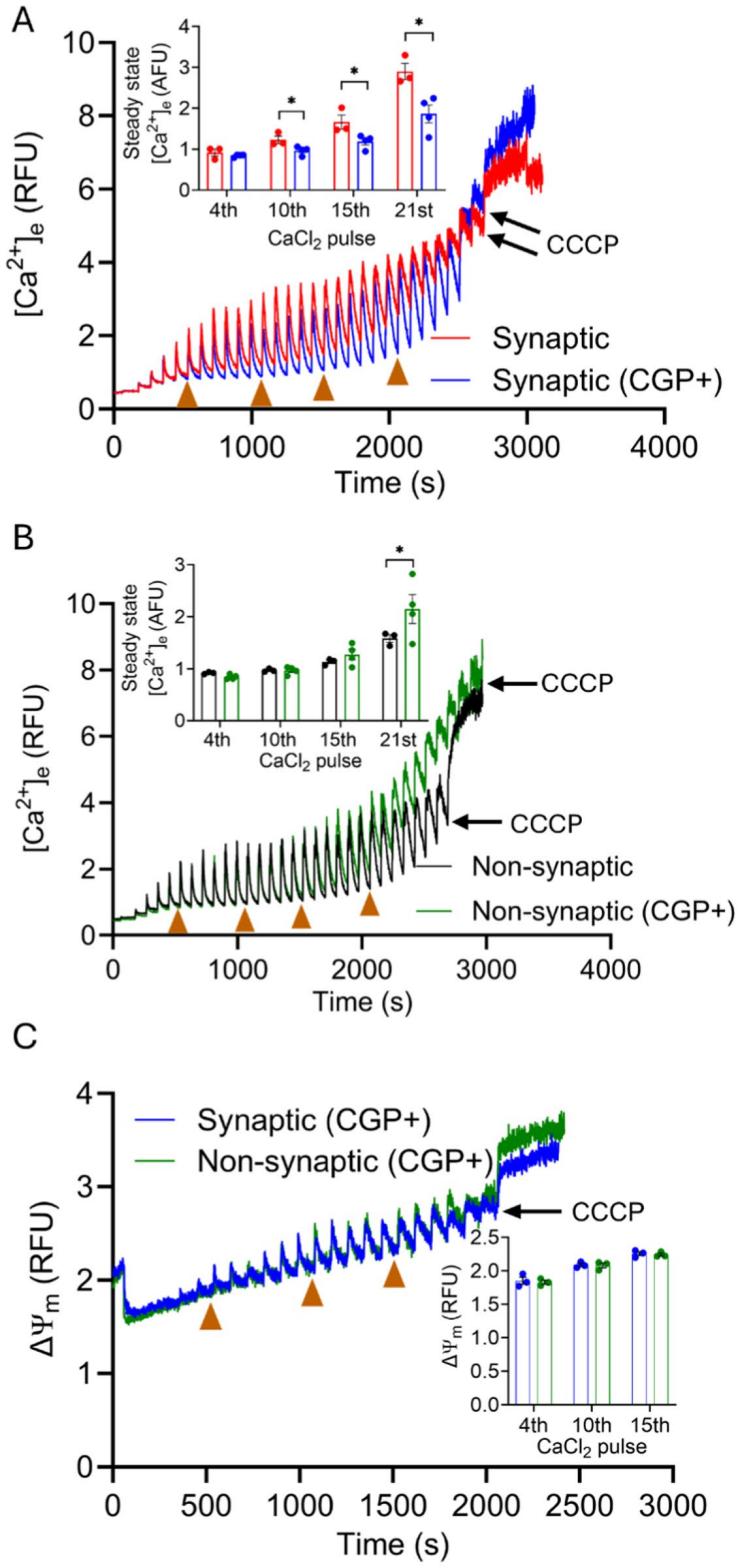
Effect of CGP on extra-mitochondrial calcium ([Ca^2+^]_e_) dynamics of synaptic and non-synaptic mitochondria. Representative traces of extra-matrix Ca^2+^ ([Ca^2+^]_e_) measured with the Ca^2+^-sensitive ratiometric dye Fura-4F in isolated synaptic **(A)** and non-synaptic **(B)** mitochondria in the presence and absence of CGP. Changes in ΔΨ_m_ in CGP-treated synaptic and non-synaptic mitochondria **(C)** measured using the ΔΨ_m_ sensitive dye TMRM (tetramethylrhodamine methyl ester perchlorate). 2 μM CGP was added in synaptic (blue trace) and non-synaptic (green trace) mitochondria at 30 s, followed by the addition of complex I substrates, [Na^+^-glutamate + Na^+^-malate (GM)] at 60 s. 40 μM CaCl_2_ pulses were added at every 90 s, and 10 μM CCCP was given at the end of the experiment. The insets show quantification of steady-state [Ca^2+^]_e_ **(A,B)** and ΔΨ_m_ **(C)** after a cumulative addition of 160, 400, 600, and 840 μM CaCl_2_. Error bars represent mean ± SEM (^*^p < 0.05).

To further evaluate these differences in mCa^2+^ handling in synaptic and non-synaptic mitochondria, we assessed the functional differences in mNCE activity. We studied the mCa^2+^ handling during boluses of CaCl_2_ in the two mitochondrial populations in the absence or presence of CGP, a specific inhibitor of mNCE (1–10 μM) (Csordas et al., 2012; Palty and Sekler, 2012; Liu et al., 2014; Islam et al., 2020; Natarajan et al., 2020). Any difference in mCa^2+^ uptake, assessed by ss[Ca^2+^]_e_ (Figures 4A,B) were attributed to Na^+^-dependent/CGP-sensitive mCa^2+^ efflux. Addition of CGP to both mitochondrial fractions energized with Na^+^-GM before CaCl_2_ pulses produced a robust mCa^2+^ uptake with [Ca^2+^]_e_ transients representing mCa^2+^ handling that was different in synaptic mitochondria *vs*. synaptic mitochondria without CGP (Figure 4A). Quantification of ss[Ca^2+^]_e_ in synaptic mitochondria showed significantly lower ss[Ca^2+^]_e_ levels, i.e., more mCa^2+^ retained, in the presence of CGP than in the absence of CGP. The inhibition of mNCE by CGP revealed that the mCa^2+^ buffering system in synaptic mitochondria is present, but its action may be secondary to the efflux system which may be dominant in mCa^2+^ handling in this population of mitochondria (Figure 4A, inset). In contrast to synaptic mitochondria, non-synaptic mitochondria did not exhibit significant differences in ss[Ca^2+^]_e_ with CGP and without CGP for most of the CaCl_2_ pulses (Figure 4B). Thus, CGP shifted the synaptic handling of excess mCa^2+^ during CaCl_2_ pulses to a pattern of mCa^2+^ dynamics that is like that of non-synaptic mitochondria without CGP.

Therefore, CGP did not significantly affect mCa^2+^ dynamics in non-synaptic mitochondria during most of the CaCl_2_ pulse challenges. Importantly, no significant differences in ΔΨ_m_ were noted in mitochondria treated with CGP in either synaptic or non-synaptic mitochondrial fractions (Figure 4C). This suggests that by inhibiting mNCE, and so blocking mCa^2+^ efflux, the excess mCa^2+^ becomes more actively buffered and sequestered so that matrix free [Ca^2+^] remains low enough to preserve ΔΨ_m_. Inhibition of mNCE-mediated Ca^2+^ extrusion to expose the sequestration of mCa^2+^ in synaptic mitochondria indicates that mNCE plays a larger role in shaping synaptic [Ca^2+^]_e_ transients than transients in non-synaptic mitochondria. Differential mCa^2+^ handling in these two populations might have physiological and pathophysiological implications.

### 3.5 Effect of altering mitochondrial matrix adenine nucleotide pool with OMN + ADP on mitochondrial Ca^2+^ handling in synaptic and non-synaptic mitochondria

**Figure 5:**
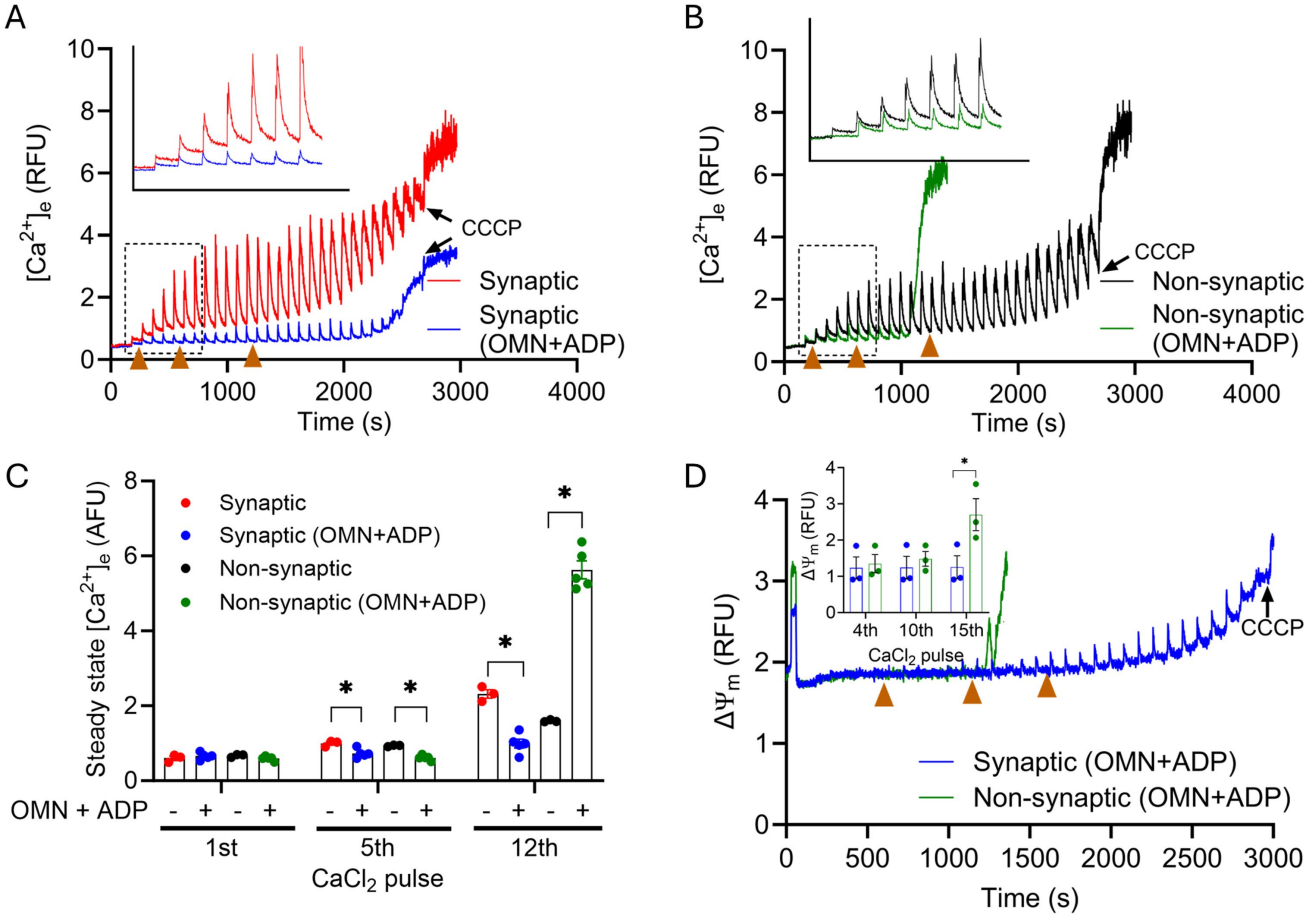
Effect of OMN + ADP on extra-mitochondrial calcium ([Ca^2+^]_e_) dynamics of synaptic and non-synaptic mitochondria. Representative traces of extra-matrix Ca^2+^ ([Ca^2+^]_e_) measured with the Ca^2+^-sensitive ratiometric dye Fura-4F in an isolated synaptic **(A)** and non-synaptic **(B)** mitochondria. 10 μM OMN and 250 μM ADP (OMN + ADP) were added in synaptic (blue trace) and non-synaptic (green trace) mitochondria at 30 s followed by the addition of the complex I substrates, [Na^+^-glutamate + Na^+^-malate (GM)] at 60 s. 40 μM CaCl_2_ pulses were added at every 90 s, and 10 μM CCCP was added at the end of each experiment. Quantification of steady-state [Ca^2+^]_e_ after a cumulative addition of 160, 400, and 600 μM CaCl_2_ **(C)**. Change in ΔΨ_m_ in OMN + ADP-treated synaptic and non-synaptic mitochondria were measured using the ΔΨ_m_ sensitive dye TMRM (tetramethylrhodamine methyl ester perchlorate) **(D)**. The insets **(A,B)** show [Ca^2+^]_m_ uptake kinetics in detail. The inset **(D)** shows ΔΨ_m_ after a cumulative addition of 160, 400, and 600 μM CaCl_2_. Error bars represent mean ± SEM (^*^p < 0.05).

Since matrix free Ca^2+^ sequestration plays an important role in shaping mCa^2+^ influx and efflux transients, we next examined whether some aspects of the mCa^2+^ buffering system contribute to differences in the Ca^2+^ handling phenotype between the two mitochondrial fractions. To do this, we again challenged synaptic and non-synaptic mitochondria as above with repeated boluses of CaCl_2,_ but in the prior presence of OMN + ADP, ADP alone, or OMN alone. We reported before (Mishra et al., 2019) in cardiomyocytes that OMN and ADP bolstered the mCa^2+^ buffering system, purportedly by modulating the matrix adenine nucleotide (AdN) pool (ADP/ATP ratio) (Haumann et al., 2010; Sokolova et al., 2013; Mishra et al., 2019). OMN + ADP given before the CaCl_2_ pulses showed rapid initial robust mCa^2+^ uptake and buffering during 7–8 pulses (280 μM–320 μM) by both synaptic and non-synaptic mitochondria, when compared to their respective untreated groups. This robust Ca^2+^ buffering was manifested by the significant diminution of the [Ca^2+^]_e_ transients and the markedly reduced ss[Ca^2+^]_e_ (Figures 5A,B).

Despite the initial strong mCa^2+^ uptake and buffering in both mitochondrial fractions, we observed, paradoxically, an early and complete collapse of ΔΨ_m_ in OMN + ADP treated non-synaptic mitochondria (Figure 5B). This observation suggests that in the non-synaptic mitochondria, the OMN + ADP-mediated matrix Ca^2+^ buffering, i.e., AdN pool, may not contribute to the protracted Ca^2+^ buffering (Figure 5A). Furthermore, if the early collapse of the ΔΨ_m_ represents mPTP opening in non-synaptic, but not in synaptic mitochondria, this appears highly unusual because a known activator of mPTP opening in isolated mitochondria is excess matrix free Ca^2+^ when the mCa^2+^ buffering system is overwhelmed. In contrast, in synaptic mitochondria, the presence of OMN + ADP resulted in prolonged and robust mCa^2+^ uptake and sequestration that endured during multiple CaCl_2_ pulse challenges (Figure 5A). Additionally, the presence of OMN + ADP strongly blunted the increase in ss[Ca^2+^]_e_ by stimulating faster mCa^2+^ uptake, sequestration, and delayed collapse of ΔΨ_m_ in synaptic mitochondria (Figure 5C). In both synaptic and non-synaptic mitochondria, the addition of ADP or OMN alone (Supplementary Figure 3) elicited a marked difference in the mCa^2+^ handling profile for both mitochondrial fractions when compared to their respective combined OMN + ADP (Figure 5) treated mitochondria. Interestingly, in non-synaptic mitochondria treated with ADP or OMN alone (Supplementary Figures 3C,D), we did not observe collapse of ΔΨ_m_ during the CaCl_2_ boluses that occurred when non-synaptic mitochondria were treated with combined OMN + ADP (Figure 5B). In addition, the fast Ca^2+^ uptake in the presence of OMN + ADP was abrogated in both synaptic and non-synaptic mitochondria. These interesting and unexpected observations portend a novel insight into the role of mCa^2+^ sequestration by the matrix adenine nucleotide pool and the impact it may have on the differential Ca^2+^ handling in these two populations of mitochondria.

Because we observed significant differences in the OMN + ADP-mediated buffering of Ca^2+^ between synaptic and non-synaptic mitochondria, we assessed again the differential effects on ΔΨ_m_ during similar CaCl_2_ pulse challenges. As expected, the addition of OMN + ADP in synaptic mitochondria displayed marked preservation of basal ΔΨ_m_ during a prolonged exposure to CaCl_2_ pulse challenges (Figure 5D). However, in the non-synaptic mitochondria, total dissipation of ΔΨ_m_ occurred early and was accompanied by maximal mCa^2+^ release into the extra-matrix space, portending mPTP opening (Figures 5B,D).

The reason for early dissipation of ΔΨ_m_ and release of mCa^2+^ in the presence of OMN + ADP in non-synaptic mitochondria is unclear. However, it is well established that excess Ca^2+^ and ROS are potent activators of mPTP opening, and they can do so by acting independently or synergistically (Chandra, 1991; Halestrap, 2010; Aldakkak et al., 2013). Thus, a possible explanation can be that the presence of OMN + ADP in non-synaptic mitochondria enhanced ROS generation (Liu and Schubert, 2009; Aklima et al., 2021) that eventually triggers early activation and opening of the mPTP. However, in the classical understanding, we did not observe mPTP opening as evidenced by the abrupt release of matrix Ca^2+^ in either population of brain mitochondria under control conditions.

**Figure 6:**
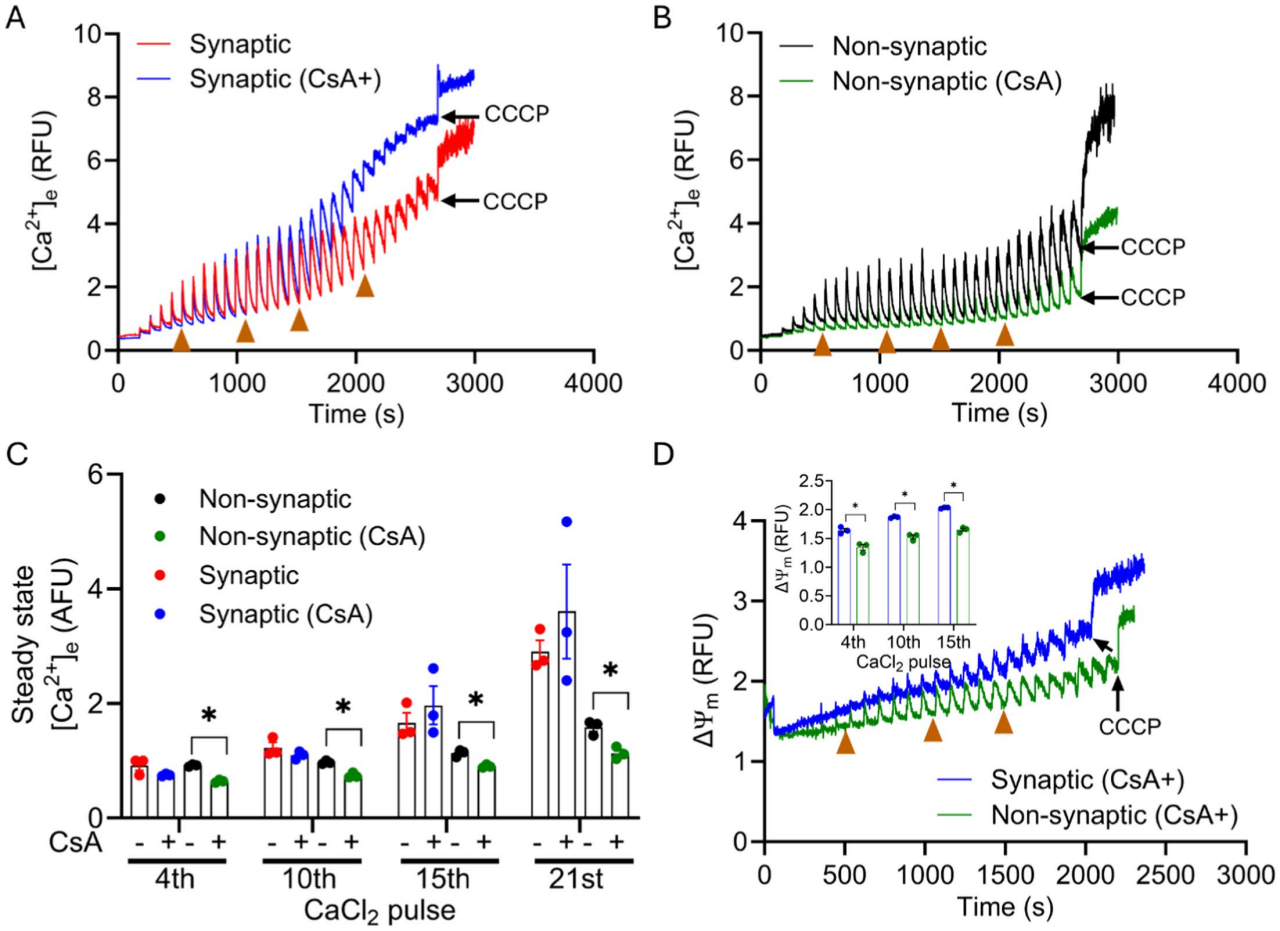
Effect of CsA on extra-mitochondrial calcium ([Ca^2+^]_e_) dynamics of synaptic and non-synaptic mitochondria. Representative traces of extra-matrix Ca^2+^ ([Ca^2+^]_e_) were measured with the Ca^2+^-sensitive ratiometric dye Fura-4FF in an isolated synaptic **(A)** and non-synaptic **(B)** mitochondria. 0.5 μM CsA was added in synaptic (blue trace) and non-synaptic (green trace) mitochondria at 30 s followed by the addition of complex I substrates, [Na^+^-glutamate + Na^+^-malate (GM)] at 60 s. 40 μM CaCl_2_ pulses were added at every 90 s, and 10 μM CCCP was added at the end of each experiment. Quantification of steady-state [Ca^2+^]_e_ after a cumulative addition of 160, 400, 600, and 840 μM CaCl_2_ **(C)**. Change in ΔΨ_m_ of OMN + ADP-treated synaptic and non-synaptic mitochondria were measured using the ΔΨ_m_ sensitive dye TMRM (tetramethylrhodamine methyl ester perchlorate) **(D)**. Inset **(D)** shows ΔΨ_m_ after a cumulative addition of 160, 400, and 600 μM CaCl_2_. Error bars represent mean ± SEM (^*^p < 0.05).

In a recent study, we reported that CsA, a potent mPTP opening inhibitor in cardiomyocytes, modulates free [Ca^2+^]_m_ by enhancing a P_i_-dependent mCa^2+^ buffering (Mishra et al., 2019). In the current study, we tested the buffering effect of CsA on [Ca^2+^]_m_ handling in synaptic and non-synaptic mitochondria (Figures 6A,B). Interestingly, in the presence of cyclosporin A (CsA), we observed more mCa^2+^ uptake in non-synaptic vs. synaptic mitochondria. The magnitude of mCa^2+^ uptake and buffering for the initial CaCl_2_ pulses, 0–900 s was similar between both groups. However, on additions of subsequent CaCl_2_ boluses, CsA treated non-synaptic mitochondria continued to show more robust mCa^2+^ uptake and sequestration with markedly lower ss[Ca^2+^]_e_ compared to untreated (control) non-synaptic mitochondria (Figures 6B,C) and synaptic mitochondria with CsA. CsA treated synaptic mitochondria, when compared to its control (untreated), exhibited less sequestration as evidenced by the gradual increase in ss[Ca^2+^]_e_ with additional CaCl_2_ pulses (Figure 6A) that was not statistically different. Thus, our result demonstrate that in the presence of CsA, non-synaptic mitochondria displayed more robust Ca^2+^ buffering with a lower ss[Ca^2+^]_e_ than in synaptic mitochondria (Figure 6B vs. Figure 6A). The magnitude of ΔΨ_m_ depolarization in both mitochondrial populations in the presence of CsA (Figure 6D) reflects distinct differences in mCa^2+^ handling, with the non-synaptic mitochondria exhibiting a more polarized ΔΨ_m_, likely because of less matrix free Ca^2+^ or greater mCa^2+^ sequestration. Taken together, the data show that CsA differentially modulates non-synaptic and synaptic mCa^2+^ handling, which is consistent with a previous observation by Naga et al. (2007), but with the potential for invoking CsA as an enhancer of matrix Ca^2+^ buffering.

### 3.6 Variable expression of mCa^2+^ handling and bioenergetics proteins in synaptic and non-synaptic mitochondria

Our results show that mitochondria from synaptic and non-synaptic brains display disparity in their bioenergetics and Ca^2+^ handling. To further elucidate the molecular underpinnings for the differential responses we examined the differences in the expression of key mitochondrial proteins involved in mCa^2+^ uptake and modulation of bioenergetics. The expression levels of the following crucial proteins were assessed in synaptic and non-synaptic mitochondrial fractions: mCU, voltage dependent anion channel 1 (VDAC1), adenine nucleotide translocase (ANT), and cyclophilin D (Cyp D). Mitochondrial Ca^2+^ uptake is primarily first through VDAC1 in the OMM, and then via the mCU in the IMM into the matrix. Additionally, VDAC1 is the main conduit for the fluxes of metabolites and nucleotides between the cytoplasm and the IMS (Camara et al., 2017). ANT, abundant in the IMM, mediates the exchange of ATP/ADP between the matrix and the IMS (Brand et al., 2005; Palmieri and Pierri, 2010). Cyp D is a mitochondrial peptidyl-prolyl cis-trans isomerase that promotes mPTP opening in the presence of excess free [Ca^2+^]_m_ (Du et al., 2008; Camara et al., 2010; Camara et al., 2011).

**Figure 7:**
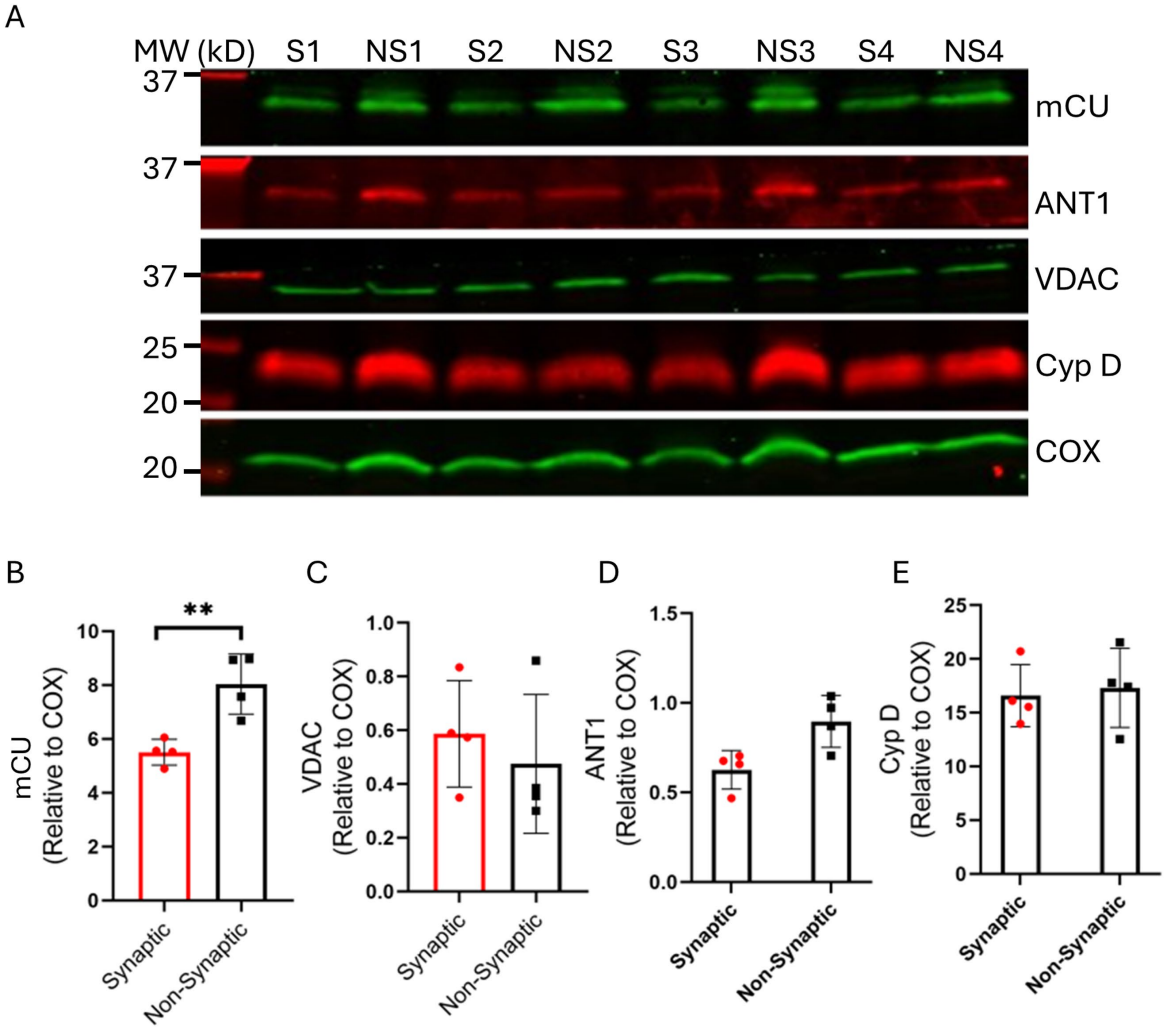
Assessment of synaptic (S) and non-synaptic (NS) mitochondrial proteins associated with mitochondrial bioenergetics and Ca^2+^ handling. Representative immunoblots **(A)** and quantification of the relative protein expressions of mCU **(B)**, VDAC **(C)**, ANT1 **(D)** and CyP D **(E)** in synaptic and non-synaptic mitochondria normalized to the mitochondrial housekeeping protein, COX IV. Error bars represent mean ± SEM (^*^p < 0.05 and ^**^p < 0.01).

We show (Figure 7) that mCU expression was higher in the non-synaptic mitochondria *vs*. synaptic mitochondria. We also show that there is no significant differences in the expression levels of VDAC1, ANT, and Cyp D between the two mitochondrial populations, when normalized to their respective mitochondrial Cox IV levels (Figure 7). We then sought to investigate expression levels of mNCE; however, we were unable to determine the true native levels because of the lack of reliable antibodies. Altogether, these results indicate that the differences in mCa^2+^ handling in the two mitochondrial populations is attributable to difference in the functional aspects of the mCa^2+^ uptake, sequestration and release, and less on the molecular levels of these proteins. These differences may have implications in the differential handling of Ca^2+^ in brain mitochondria during transient and cellular Ca^2+^ overload.

## 4 Discussion

In the brain, the large number of different cell types, with distinct metabolic profiles, excitability, and ion channel composition, contribute to the heterogeneity of brain function. Different brain cell mitochondria also exhibit a high degree of functional heterogeneity, depending on the specific cell type (e.g., mitochondria from neurons *vs*. glial cells) or subcellular locations within the neuron (i.e., soma, dendrites, axons and synapses) (Sonnewald et al., 1998; Pekkurnaz and Wang, 2022; Chen et al., 2023). The regional or local mitochondrial phenotypic differences are found not to consist of more variability in their mitochondrial content, but more variability in their utilization for ATP synthesis and specialization for tasks in different brain cell types (Mosharov et al., 2024). In so far as cCa^2+^ signaling is a key regulator of these cell functions, this functional heterogeneity may reflect how mitochondria from different brain cells or even within the same cell, display differential sensitivities to metabolism, stress, and trauma based on their differences in mCa^2+^ handling (Kulbe et al., 2017; Hill et al., 2018; Pekkurnaz and Wang, 2022).

Our goal in this study was to provide a systematic comparison of the dynamics of Ca^2+^ handling in synaptic and non-synaptic mitochondria from rat brain. Although several studies have examined and reported on the heterogeneity in the Ca^2+^ handling of synaptic and non-synaptic mitochondria (Brown et al., 2006; Naga et al., 2007), those studies did not delve into a detailed examination of mCa^2+^ dynamics in these two mitochondrial populations. In this study, we: (1) monitored basal mitochondrial bioenergetics and energetic efficiency, and (2) characterized mCa^2+^ handling, specifically mCa^2+^ uptake, buffering (sequestration), and extrusion in synaptic and non-synaptic mitochondria.

Our key functional findings are: (i) the rate of state 3 (ADP-stimulated) respiration is similar between the two populations of mitochondria. However, state 4 (ADP-depleted) respiration was faster in non-synaptic than in synaptic mitochondria, which results in a lower RCI in non-synaptic mitochondria; (ii) basal ΔΨ_m_ remains the same before and during CaCl_2_ boluses between the two populations; (iii) non-synaptic mitochondria take up and sequestered more Ca^2+^ than synaptic mitochondria consistent with prior reports (Brown et al., 2006; Naga et al., 2007); (iv) synaptic mitochondria exhibit higher mNCE-mediated Ca^2+^ efflux activity than non-synaptic mitochondria; (v) synaptic and non-synaptic mitochondria exhibit two distinct modes of matrix Ca^2+^ buffering: (a) enhanced and protracted matrix Ca^2+^ buffering by AdN pool (OMN + ADP) in synaptic vs. non-synaptic mitochondria, and (b) enhanced and protracted Ca^2+^ buffering by CsA in non-synaptic vs. synaptic mitochondria. Our quantitative protein assay demonstrate that: (i) there is greater mCU expression in non-synaptic vs. synaptic mitochondria, and (ii) there are no significant differences between the two populations in expression of VDAC1, ANT, and Cyp D proteins. Taken together, our study reveals differential mCa^2+^ handling features unique to synaptic and non-synaptic mitochondria that might be central to understanding their functional differences in the brain, and that there are potential implications for differential susceptibility to injury (Kulbe et al., 2017; Hill et al., 2018), particularly when this heterogeneity results in differential cCa^2+^ and mCa^2+^ overload in different brain areas.

### 4.1 Basal mitochondrial bioenergetics in synaptic and non-synaptic mitochondria

Synaptic mitochondria are isolated from viable synaptosomes, while non-synaptic mitochondria are isolated from various brain cell types, including glia and soma of neurons (Naga et al., 2007). The two populations of mitochondria display distinct morphological (Lewis et al., 2018; Faitg et al., 2021) and biochemical features (Lai et al., 1977; Graham et al., 2017), as well as differential vulnerabilities to oxidative damage (Banaclocha et al., 1997; Lores-Arnaiz and Bustamante, 2011; Hill et al., 2018) and Ca^2+^ overload (Brown et al., 2006; Naga et al., 2007; Yarana et al., 2012). To ascertain that the two populations of mitochondria accurately represent synaptic and non-synaptic mitochondria, we conducted western blot studies and showed synaptophysin and PSD95, distinct molecular markers of synaptic mitochondria, were markedly expressed in synaptic, but not in non-synaptic mitochondria (Figure 1). We showed that the isolated mitochondrial fractions were: (1) fractions of comparable purity (Figure 1), (2) analytical grade for key protein markers expression (Figure 7), and (3) functionally competent for bioenergetics and Ca^2+^ handling studies (Figures 2–6).

Heterogeneity in brain mitochondrial respiratory function has been studied and is due to the different functions and metabolism of different brain cell types (Gilmer et al., 2010a, 2010b; Kulbe et al., 2017). Synaptic mitochondria exhibit either decreased O_2_ consumption rates compared to non-synaptic mitochondria (Gilmer et al., 2010a, 2010b; Kulbe et al., 2017) or no significant difference in respiratory capacity (Hamberger et al., 1970; Hamilton et al., 2021). We found that synaptic mitochondria display a more coupled respiration, i.e., RCI than non-synaptic mitochondria. This occurred even though both mitochondrial populations exhibited comparable basal (state 2), and ADP stimulated (state 3) respirations, whereas non-synaptic mitochondria exhibited a higher state 4 respiration (after total ADP phosphorylation), likely due to a mild H^+^ leak, and hence the lower RCI. Mild uncoupling, due to an increase in H^+^ leak, stimulates respiration to maintain ΔΨ_m_. Oligomycin is a robust and definitive measure of state 4 respiration because it blocks any ATP formation. However, rather than using oligomycin to expose a residual H^+^ leak in the two mitochondrial populations we used depletion of ADP as a marker of ceased ATP production to assess H^+^ leak during state 4. Nevertheless, it is unclear why we see the non-synaptic mitochondria display increased state 4 H^+^ leak that leads to the mild uncoupling.

### 4.2 Differential mechanisms of mCa^2+^ handling in synaptic and non-synaptic mitochondria

Ca^2+^ homeostasis is important for regulating the release of neurotransmitters at synaptic terminals and for the required ATP supply to execute these functions. As excitable cells, neurons, especially at the synaptic terminal, have high fluxes of Ca^2+^ in and out of their mitochondria that is coupled to their high energetic demand (Kessinger et al., 1991; Li et al., 2004) for neurotransmission. Compared to synaptic mitochondria, non-synaptic mitochondria are reported to be less sensitive to mCa^2+^ overloading (Brown et al., 2006; Naga et al., 2007). Here, we examined in more detail the changes in mCa^2+^ dynamics, in synaptic and non-synaptic mitochondria, during transient pulses of CaCl_2_ in the presence or absence of drugs with known effects to alter mCa^2+^ uptake, sequestration, or efflux.

Consistent with prior reports (Brown et al., 2006; Naga et al., 2007), we found a marked difference in mCa^2+^ handling between synaptic and non-synaptic mitochondria (Figure 3). We used measured, intermittent boluses of CaCl_2_ to have a better appreciation of the detailed mechanisms of mCa^2+^ handling in the two mitochondrial fractions. In both populations of mitochondria, as CaCl_2_ is added to the mitochondrial suspension, the extra-matrix Ca^2+^ signal rapidly and transiently increases, and then decreases as the Ca^2+^ disappears into the mitochondrial matrix via mCU. The nadir at each pulse interval and after Ca^2+^ uptake represents the extra-matrix steady-state (ss[Ca^2+^]_e_) attained once the bolus of CaCl_2_ enters the matrix and becomes sequestered. In a recent study (Mishra et al., 2019), we defined ss[Ca^2+^] as largely attributable to the buffering capacity of mitochondria for Ca^2+^. This ss[Ca^2+^] was determined to be inversely proportional to mCa^2+^ buffering capacity (Mishra et al., 2019). In the present study, while mitochondria were challenged with more CaCl_2_ pulses, non-synaptic mitochondria continued to markedly take up and buffer the exogenous CaCl_2_ for an extended period, while synaptic mitochondria buffered less of the added CaCl_2_. Thus, in synaptic mitochondria, the added CaCl_2_ gradually accumulated in the external buffer, resulting in a higher ss[Ca^2+^] than in non-synaptic mitochondria.

We posit that one of two mechanisms could contribute to the increase ss[Ca^2+^]_e_: (1) less mCa^2+^ uptake due to decrease in the ΔΨ_m_, with a corresponding decrease in the driving force for mCU-mediated mCa^2+^ uptake; or (2) extrusion of the mCa^2+^ via the mNCE if buffering mechanisms are inadequate or overridden. Although mCU expression is higher in non-synaptic mitochondria (Figure 7), the similarity in ΔΨ_m_ (Figure 3) and the Ca^2+^ decay constant (Supplementary Figure 2) during the CaCl_2_ pulse challenges in both mitochondrial fractions suggest no differences in mCa^2+^ uptake contributed to the higher ss[Ca^2+^]_e_ in synaptic vs. non-synaptic mitochondria. We reasoned that the mCa^2+^ levels for the two mitochondrial populations were maintained, in part, by different mechanisms that regulate sequestration and/or efflux.

The capacity of mitochondria to sequester mCa^2+^ depends on the amount of mCa^2+^ uptake and mCa^2+^ release. Thus, the matrix-free Ca^2+^ reflects the Ca^2+^ fluxes across the IMM and the matrix buffering. We found that the higher ss[Ca^2+^]_e_ levels in synaptic mitochondria are more likely attributed to increased mNCE activity, resulting in greater mCa^2+^ release of free mCa^2+^. This notion of a slow release of Ca^2+^ from mitochondria leading to a gradual increase in ss[Ca^2+^]_e_ has also been proposed in brain mitochondria during exposure to multiple boluses of CaCl_2_ (Hamilton et al., 2021). To test if the increased Ca^2+^ efflux is mediated by active mNCE during the CaCl_2_ boluses, we added CGP, a mNCE inhibitor, to both mitochondrial suspensions before adding CaCl_2_. The presence of CGP markedly increased mCa^2+^ uptake and mCa^2+^ sequestration in synaptic mitochondria as evidenced by lowering of the ss[Ca^2+^]_e_ to levels observed in non-synaptic mitochondria without CGP. The extended robust uptake of mCa^2+^ and the lower ss[Ca^2+^]_e_ in synaptic mitochondria in the presence of CGP suggest that mNCE is more active in synaptic mitochondria *vs*. non-synaptic mitochondria. This differential activity of mNCE could lead to extrusion of some of the added Ca^2+^ in synaptic mitochondria. In non-synaptic mitochondria, mCa^2+^ handling with CGP was like that of non-CGP treated non-synaptic mitochondria for most of the duration of the CaCl_2_ pulse challenges (Figure 4). Furthermore, as there was no significant change in non-synaptic uptake and buffering of mCa^2+^ with CGP for most of the Ca^2+^ pulse challenges, this suggests there is a reduced or negligible mNCE role to regulate [Ca^2+^]_m_ during CaCl_2_ bolus challenges, compared with synaptic mitochondria. Thus, synaptic mitochondria likely maintain free mCa^2+^ by an active mNCE-mediated Ca^2+^ extrusion pathway rather than by mCa^2+^ buffering and sequestration during CaCl_2_ pulse challenge.

In comparison, non-synaptic mitochondria did not respond to CGP in regulating their mCa^2+^ level via a mNCE-mediated Ca^2+^ extrusion pathway. This heterogeneity in mCa^2+^ handling may reflect differential mechanisms in intracellular Ca^2+^ signaling in synaptic and non-synaptic regions of the brain. Impaired mNCE activity has been associated with reduced synaptic activity and mental retardation (Stavsky et al., 2021; Cabral-Costa et al., 2023); in addition, hippocampal neuron-specific deletion of NCLX, aka mNCE, was reported to impair cognitive performance (Jadiya et al., 2023). On the contrary, *in vivo* genetic deletion of the mNCE in hippocampal astrocytes was associated with improved cognitive performance in behavioral tasks (Cabral-Costa et al., 2023). Other studies report reduced cCa^2+^ levels following mNCE inhibition (Palty et al., 2010; Cabral-Costa et al., 2023; Ramos et al., 2024). Based on our results, we postulate that mNCE function is the primary regulator at synaptosomes, and that buffering acts only as a secondary regulator of mCa^2+^ homeostasis when mNCE is inactive during cCa^2+^ overload. In this case, the mNCE inhibition would contribute to greater cCa^2+^ dysregulation, unless the mCa^2+^ buffering system is activated to preserve cCa^2+^ and mCa^2+^ homeostasis.

Our premise is that the net ss[Ca^2+^]_e_ during each bolus of CaCl_2_ is reflective of the aggregate amount of Ca^2+^ uptake, buffered and/or released. We have posited that maintenance of a low ss[Ca^2+^]_e_ is in large part attributable to increased sequestration of mCa^2+^ by the buffering of Ca^2+^ with phosphates in the matrix. Indeed, the significance of dynamic mCa^2+^ buffering and the role of phosphate and other factors in regulating matrix free Ca^2+^ has been described in detail in our previous studies (Blomeyer et al., 2013; Boelens et al., 2013; Haumann et al., 2018; Mishra et al., 2019). The accumulation of nucleotides ADP + ATP in the matrix has been implicated in delaying mCa^2+^-induced mPTP opening (Hunter and Haworth, 1979; Halestrap et al., 1997). In an earlier study in cardiomyocytes, we found that adding ADP increased measured free [Ca^2+^]_m_ transiently, probably by removing matrix [P_i_] when ADP + P_i_ is converted to ATP, which is then removed from the matrix. In the presence of OMN, an ATP synthase (complex V) inhibitor, the transient increase in [Ca^2+^]_m_ is inhibited because ADP + Pi cannot be converted to ATP (Haumann et al., 2010). Altering the adenine nucleotide (AdN) pool is consistent with our recent findings where we showed OMN + ADP bolstered cardiomyocyte mCa^2+^ buffering capacity (Mishra et al., 2019). We proceeded to confirm further whether the differences in Ca^2+^ handling between synaptic and non-synaptic mitochondria are due to differences in their mCa^2+^ buffering capabilities. To achieve this, we treated mitochondria with OMN + ADP or with CsA, two conditions we have shown enhance matrix Ca^2+^ buffering in cardiomyocytes (Mishra et al., 2019).

Because our current data show that matrix Ca^2+^ buffering in the synaptic mitochondria is bolstered by OMN + ADP, this suggests that in the synaptic mitochondria, AdNs may play a role in matrix Ca^2+^ buffering during exposure to excess Ca^2+^. Interestingly, the magnitude of mCa^2+^ uptake and retention are more pronounced in synaptic mitochondria in the presence of OMN + ADP than any other condition (Figure 5A). The matrix Ca^2+^ buffering in synaptic mitochondria in the presence of OMN + ADP was associated with normal basal ΔΨ_m_ (Figure 5D) during the CaCl_2_ bolus protocol. These observations suggest that the matrix buffering, which is supplemented by the active mNCE, is partly dependent on the AdN pool. In contrast, in non-synaptic mitochondria, the presence of OMN + ADP paradoxically caused early dissipation of the ΔΨ_m_ and mCa^2+^ release (Figures 5B,D), even after initially showing robust mCa^2+^ uptake and sequestration. This apparent worsening of the mCa^2+^ handling in non-synaptic mitochondria in the presence of OMN + ADP suggests that the buffering of mCa^2+^ under control conditions (Figure 2) is not mediated by an AdN-dependent buffering mechanism, but by a different buffering means. This notion is supported by our supplemental data that show in non-synaptic mitochondria, ADP and OMN given alone, led to less mCa^2+^ retention as evidenced by higher ss[Ca^2+^]_e_ and unstable mCa^2+^ dynamics (Supplementary Figures 3C,D). In contrast, in synaptic mitochondria both ADP and OMN given alone before the first CaCl_2_ pulse showed improved ss[Ca^2+^]_e_ (Supplementary Figures 3A,B) compared to their untreated controls (Figure 2A). The early collapse of the ΔΨ_m_ and concomitant release of mCa^2+^ despite some initial rapid uptake and buffering of the Ca^2+^ in the non-synaptic mitochondria is baffling but experiments to understand this are beyond the scope of our study.

### 4.3 CsA enhances matrix Ca^2+^ sequestration more in non-synaptic than in synaptic mitochondria

Our results also demonstrate that non-synaptic mitochondria rely on an unidentified matrix buffering system that is not mediated by ADP+ ATP to handle the protracted CaCl_2_ pulse challenges. So, we explored the possible mCa^2+^ buffering mechanisms. According to a previous report from our group (Blomeyer et al., 2013), at least two dynamic classes of mCa^2+^ buffers are known for Ca^2+^ buffering (Bazil et al., 2013). We propose that one class of buffers bind a single Ca^2+^ ion at a single binding site, i.e., like classical Ca^2+^ buffers (Coll et al., 1982; Corkey et al., 1986). Another class of buffer is associated with formation of amorphous Ca^2+^-phosphates, which have the potential of binding multiple Ca^2+^ ions at a single site in a cooperative fashion (Bazil et al., 2013). Moreover, we reported recently that CsA maintains low free [Ca^2+^]_m_ in cardiomyocytes, in part by stimulating and/or potentiating a P_i_-dependent matrix Ca^2+^ buffering system (Mishra et al., 2019).

To investigate further the potential contribution of this buffering system in synaptic *vs.* non-synaptic mCa^2+^ handling, CsA was added to the mitochondrial suspensions before the CaCl_2_ pulses. Interestingly, unlike OMN + ADP, CsA enhanced mCa^2+^ sequestration as evidenced by the marked reduction in ss[Ca^2+^]_e_ in non-synaptic mitochondria, but not so well in synaptic mitochondria (Figure 6). The differential responses indicate that the non-synaptic and synaptic mitochondria constitutes different classes of matrix Ca^2+^ buffering. Furthermore, these differential responses to the buffering factors and their impact on mCa^2+^ handling have implications for maintaining [Ca^2+^]_m_ homeostasis in non-synaptic *vs.* synaptic mitochondria. However, additional studies are needed to delve further into the nature of the Ca^2+^ buffering systems in these two mitochondrial populations and to delineate the underlying buffering mechanisms in brain mitochondria relevant to understanding Ca^2+^ regulation at the synapses.

### 4.4 Atypical or absent mPTP opening in synaptic and non-synaptic mitochondria during CaCl_2_ pulse challenges

In our study, both synaptic and non-synaptic mitochondria, under control conditions, did not display the characteristic mPTP opening during CaCl_2_ pulse challenges observed in mitochondria isolated from cardiomyocytes (Aldakkak et al., 2011; Mishra et al., 2019; Mishra and Camara, 2022). This lack of distinct mPTP opening and absence of massive extrusion of mCa^2+^, as shown by the rapid increase in the extra-matrix fluorescent signal for [Ca^2+^]_e_, is consistent with other studies using isolated brain mitochondria (Andreyev et al., 1998; Berman et al., 2000).

Biophysically, classic mPTP opening is characterized by matrix swelling and a dramatic increase in IMM permeability, with the subsequent release of mCa^2+^, and accompanied by a total collapse of ΔΨ_m_ and release of pro-apoptotic factors like cytochrome *c* (Bernardi et al., 1992; Szabo and Zoratti, 1992; Basso et al., 2005; Mishra et al., 2019; Sun et al., 2022). Unlike in other tissues like heart (Mishra et al., 2019; Sun et al., 2022) and liver (Varanyuwatana and Halestrap, 2012; Kim et al., 2023), we found that repetitive boluses of CaCl_2_ to both synaptic and non-synaptic mitochondria only led to gradual increase in ss[Ca^2+^]_e_, without IMM permeabilization, massive release of matrix Ca^2+^ or complete loss of ΔΨ_m_. So, we posit that in contrast to heart mitochondria where we observe mPTP opening with lower [Ca^2+^] (Natarajan et al., 2020), brain mitochondria, with higher [Ca^2+^], show a much greater capacity to sequester Ca^2+^, and or to extrude Ca^2+^ leading to more Ca^2+^ uptake. This is evidenced by the progressive increase in matrix Ca^2+^ in non-synaptic mitochondria, and the continuing decrease in ss[Ca^2+^]_e_ in synaptic mitochondria with similar gradual dissipations of ΔΨ_m_ in the two mitochondrial population. This gradual depolarization leads eventually to the diminution of the driving force for further Ca^2+^ uptake and thereby prevents the matrix from reaching the critical Ca^2+^ threshold required to open the pore. Another possible explanation is that the mPTP may constitute different entities in brain and heart cells. In concordance with this notion, Bernardi et al. (2023) argued that “remaining issues” in the current debate about the structure and identity of the pore include defining whether there is more than one protein type of mPTP.

In our study, a sudden spike in mCa^2+^ release or ΔΨ_m_ depolarizations at the end of each protocol were caused by the application of CCCP; this was given to establish maximal ΔΨ_m_ depolarization and release of some of the matrix Ca^2+^. An interesting, and unexplainable finding, is that only in the presence of OMN + ADP did both synaptic and non-synaptic mitochondria exhibit collapse of ΔΨ_m_ and total release of mCa^2+^ during the CaCl_2_ pulse challenges. These events occurred much earlier in non-synaptic mitochondria compared with synaptic mitochondria (Figure 5). As alluded to previously, these unexpected results in the presence of OMN + ADP suggest yet identified features in the constituents of the mPTP complex in different tissues.

### 4.5 Potential molecular factors contributing to the differential Ca^2+^ handling and differences in bioenergetics in synaptic *vs*. non-synaptic mitochondria

Molecular mechanisms could contribute to the differences in mCa^2+^ handling between synaptic vs. non-synaptic mitochondria. To supplement physiological studies, we examined key mitochondrial proteins involved in regulating [Ca^2+^]_m_. Consistent with a previous study (Stauch et al., 2014), western blot showed lower expression of mCU in synaptic *vs*. non-synaptic mitochondria (Figure 7). Knockdown of endogenous mCU expression leads to reduction of NMDA-mediated increase in mCa^2+^, lower levels of ΔΨ_m_ depolarizations and prevents excitotoxicity (Qiu et al., 2013). Since synaptic mitochondria are reported to be more susceptible to Ca^2+^ overload (Brown et al., 2006; Naga et al., 2007; Yarana et al., 2012), the lower expression of mCU suggests less Ca^2+^ uptake, and concomitantly, less [Ca^2+^]_m_ overload during transient increases in cCa^2+^. However, whether the differences in the expression of mCU contributed significantly to the differences in mCa^2+^ handling between the synaptic and non-synaptic mitochondria is unclear, since the pore-forming mCU is regulated by a complex of associated proteins (MacEwen and Sancak, 2023). Furthermore, based on similarities of ΔΨ_m_ profiles, mCa^2+^ uptake (Supplementary Figure 2) and buffering in the presence and absence of CGP in synaptic and non-synaptic mitochondria, respectively, we propose that the uptake phase of mCa^2+^ may not contribute to the differential regulation of [Ca^2+^]_m_ in these two fractions.

There was no significant difference in the expressions of VDAC1, ANT and Cyp D between synaptic and non-synaptic mitochondria (Figure 7). The lack of differences in VDAC1 on the OMM indicate that both populations likely have a similar [Ca^2+^]_m_ in their IMS during the CaCl_2_ pulse challenges. Similarities in ANT expression suggest no significant differences in the translocation of ADP and ATP across the IMM of the two mitochondrial fractions. A previous study (Naga et al., 2007) showed significant differences in the expression of Cyp D between the two populations, with synaptic mitochondria showing higher Cyp D expression. Increased Cyp D expression sensitizes mitochondria to Ca^2+^-mediated mPTP opening (Du et al., 2008; Camara et al., 2010). CsA binds to mitochondrial Cyp D and so may desensitize mitochondria to excess Ca^2+^ (Hamilton et al., 2021) or may enhance matrix Ca^2+^ sequestration (Mishra et al., 2019). However, in our study, we observed no significant differences in Cyp D expression between the two populations. Moreover, the presence of CsA bolstered the mCa^2+^ uptake and buffering in non-synaptic *vs*. synaptic mitochondria. These findings are inconsistent with reports that show more CsA was required in synaptic mitochondria to inhibit mPTP. This inconsistency may be ascribed to the lack of significant differences in CypD between the two fractions in our study. This suggests that in our study the CsA effect on mCa^2+^ could be attributable to its P_i_-dependent buffering effect, as we reported previously (Mishra et al., 2019).

Overall, our result indicate that the mNCE likely plays a prominent role in maintaining free [Ca^2+^]_m_ in synaptic mitochondria by ejecting much of the Ca^2+^ during periods of increased extra-matrix CaCl_2_ challenges. Regarding the role of mNCE activity between the two mitochondrial fractions, we postulate that it could, in part, be attributed to increased expression of the exchanger. Unfortunately, we were unable to confirm this notion because of the lack of reliable mNCE antibodies at the time. Other contributing factors to the high mNCE -mediated Ca^2+^ efflux could be increased activity through posttranslational modification (PTM) of the exchanger (Kostic et al., 2015). Indeed, there is evidence from a recent report that mNCE activity is increased via a PKA-mediated phosphorylation of the exchanger. This targeted phosphorylation is reported to reverse [Ca^2+^]_m_ overload by increasing mCa^2+^ efflux and to promote cell survival (Kostic et al., 2015).

PTM studies are beyond the scope of our study and are also hampered by the lack of reliable commercially available phospho-mNCE antibodies. Nonetheless, delineating these molecular mechanisms could provide novel insights into our understanding of the function of the mNCE in preserving Ca^2+^ homeostasis during periods of increased synaptic Ca^2+^ transients during neurotransmission at the synapse. Lastly, mNCE function, which is electrogenic, is dependent on ΔΨ_m_, as we and others have reported (Blomeyer et al., 2013; Blomeyer et al., 2016; Kostic et al., 2018); however, we found no difference in basal ΔΨ_m_ in synaptic and non-synaptic mitochondria. In addition, the transient and gradual depolarization of ΔΨ_m_ during the CaCl_2_ pulse challenges were similar for the two populations, suggesting that the mNCE was not hampered by ΔΨ_m_. Altogether, these results indicate that synaptic and non-synaptic mitochondria employ different strategies to cope with the challenges of excess [Ca^2+^]_m_ to sustain their resting ΔΨ_m_, that is essential to regulate Ca^2+^ homeostasis.

## 5 Summary and conclusion

Our study demonstrates that synaptic and non-synaptic mitochondria handle excess mCa^2+^ by predominately separate mechanisms that likely involve dependencies on mCa^2+^-induced extrusion via mNCE vs. mCa^2+^ buffering, respectively. The reliance on Ca^2+^ efflux in synaptic mitochondria and Ca^2+^ buffering in non-synaptic mitochondria, combined with no significant differences in ΔΨ_m_ between the two populations during the CaCl_2_ pulse challenges confirm that different mechanisms are involved in regulating [Ca^2+^]_m_ during mCa^2+^ overload. In synaptic mitochondria, the quiescent and nominal matrix buffering is dependent, in part, on AdN (OMN + ADP)-mediated mCa^2+^ sequestration. In contrast, the active matrix buffering in the non-synaptic mitochondria is dependent, in part, on P_i_-mediated mCa^2+^ buffering system that is bolstered by CsA. The implication of these observations may portend differences in the mCa^2+^ buffering systems and the regulation of ΔΨ_m_ in these two populations of mitochondria.

Altogether, our study provides new mechanistic insights into how synaptic and non-synaptic mitochondria differentially handle Ca^2+^ during exposure to excess extra-matrix Ca^2+^. Synaptic mitochondria are susceptible to Ca^2+^ dysregulation during cCa^2+^ overload (Brown et al., 2006; Naga et al., 2007; Yarana et al., 2012) and oxidative stress (Banaclocha et al., 1997; Lores-Arnaiz and Bustamante, 2011; Hill et al., 2018), as reported in some neurological disorders, like traumatic brain injury (Kulbe et al., 2017; Hill et al., 2018) and other neurodegenerative diseases (Du et al., 2012). Therefore, this in-depth assessment of ss[Ca^2+^]_e_ on cCa^2+^ homeostasis in synaptic and non-synaptic mitochondria could contribute to our understanding of the potential roles of these two brain mitochondrial populations in the etiology and progression of neurodegenerative diseases.

## Data Availability

The original contributions presented in the study are included in the article/Supplementary material, further inquiries can be directed to the corresponding author.

## Supplementary material

SUPPLEMENTARY FIGURE 1 Respiration in isolated mitochondria from synaptic and non-synaptic fraction. State 2 **(A)**, 3 **(B)** and 4 **(C)** respiration with the complex I substrates, [Na^+^-glutamate + Na^+^-malate (GM)]. Error bars represent mean ± SEM (^*^*p* < 0.05 and ^**^*p* < 0.01).

SUPPLEMENTARY FIGURE 2 Representative traces of extra-matrix Ca^2+^ ([Ca^2+^]_e_) measured with the Ca^2+^-sensitive ratiometric dye Fura-4FF. Inset shows quantification of decay constants. Error bars represent mean ± SEM.

SUPPLEMENTARY FIGURE 3 Effect of ADP alone **(A,C)** and OMN alone **(B,D)** on extra-mitochondrial calcium ([Ca^2+^]_e_) dynamics of synaptic **(A,B)** and non-synaptic **(C,D)** mitochondria. Representative traces of extra-matrix Ca^2+^ ([Ca^2+^]_e_) measured with the Ca^2+^-sensitive ratiometric dye Fura-4F in isolated synaptic **(A,B)** and non-synaptic **(C,D)** mitochondria. 250 μM ADP **(A,C)** and 10 μM OMN **(B,D)** were added to synaptic (pink traces) and non-synaptic (gray traces) mitochondria at 30 s followed by the addition of the complex I substrates, [Na^+^-glutamate + Na^+^-malate (GM)] at 60 s. 40 μM CaCl_2_ pulses were added at every 90 s, and 10 μM CCCP was added at the end of each experiment.
