# Supplementary figures and images for: Differential Ca2+ handling by isolated synaptic and non-synaptic mitochondria: roles of Ca2+ buffering and efflux
Source: Front Synaptic Neurosci. 2025 May 27;17:1562065. doi: 10.3389/fnsyn.2025.1562065 (PMC12149423; doi:10.3389/fnsyn.2025.1562065)

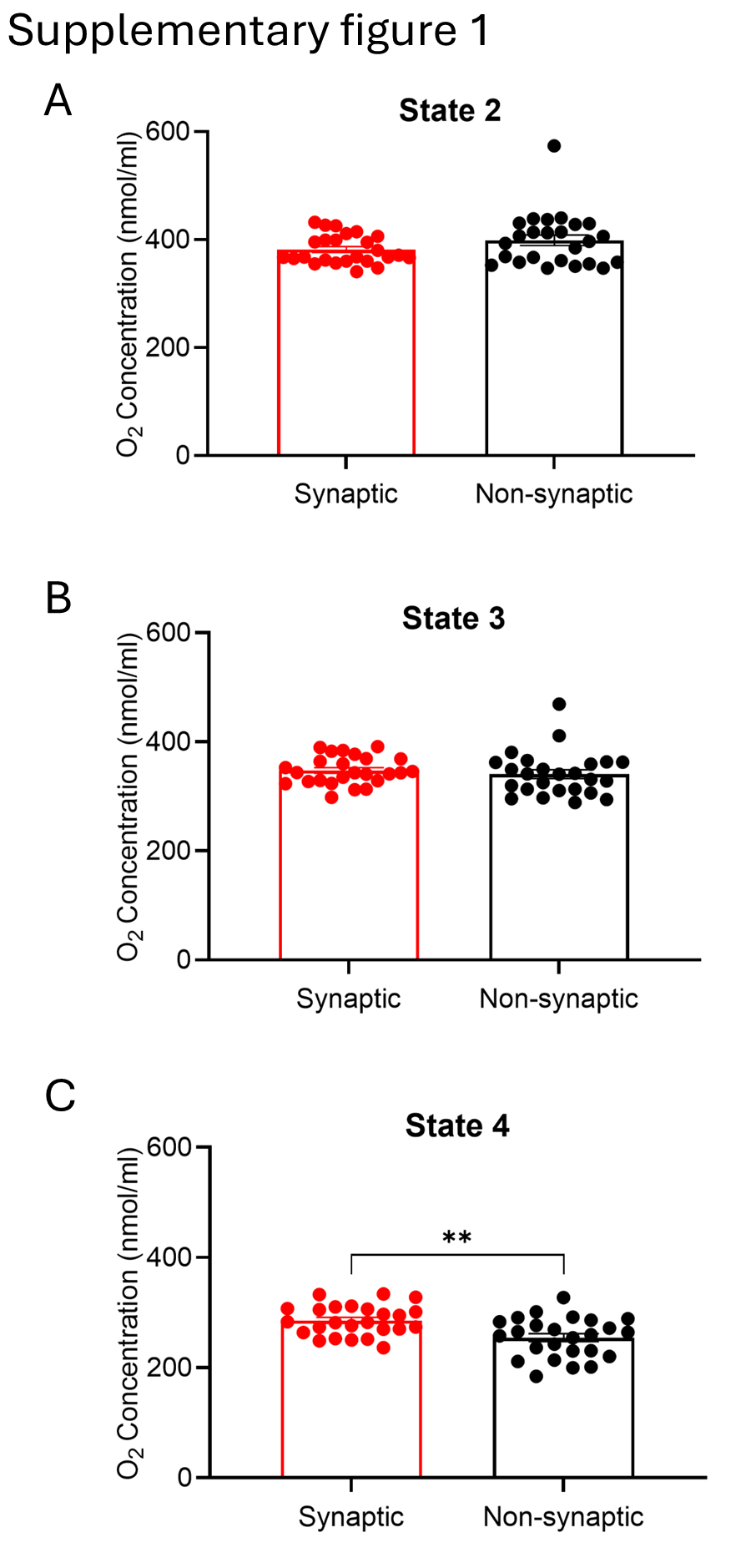

Supplement: SUPPLEMENTARY FIGURE 1 — Respiration in isolated mitochondria from synaptic and non-synaptic fraction. State 2 (A), 3 (B) and 4 (C) respiration with the complex I substrates, [Na+-glutamate + Na+-malate (GM)]. Error bars represent mean ± SEM (*p < 0.05 and **p < 0.01). [file Image_1.TIF]

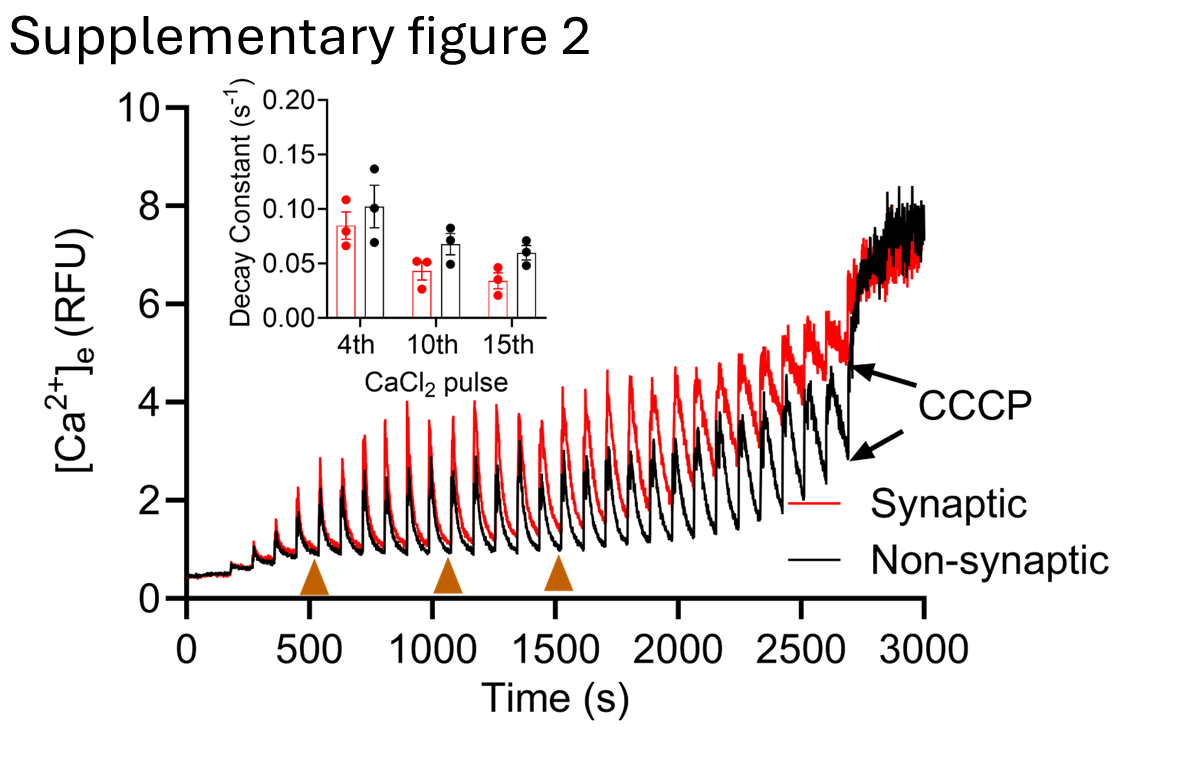

Supplement: SUPPLEMENTARY FIGURE 2 — Representative traces of extra-matrix Ca2+ ([Ca2+]e) measured with the Ca2+-sensitive ratiometric dye Fura-4FF. Inset shows quantification of decay constants. Error bars represent mean ± SEM. [file Image_2.TIF]

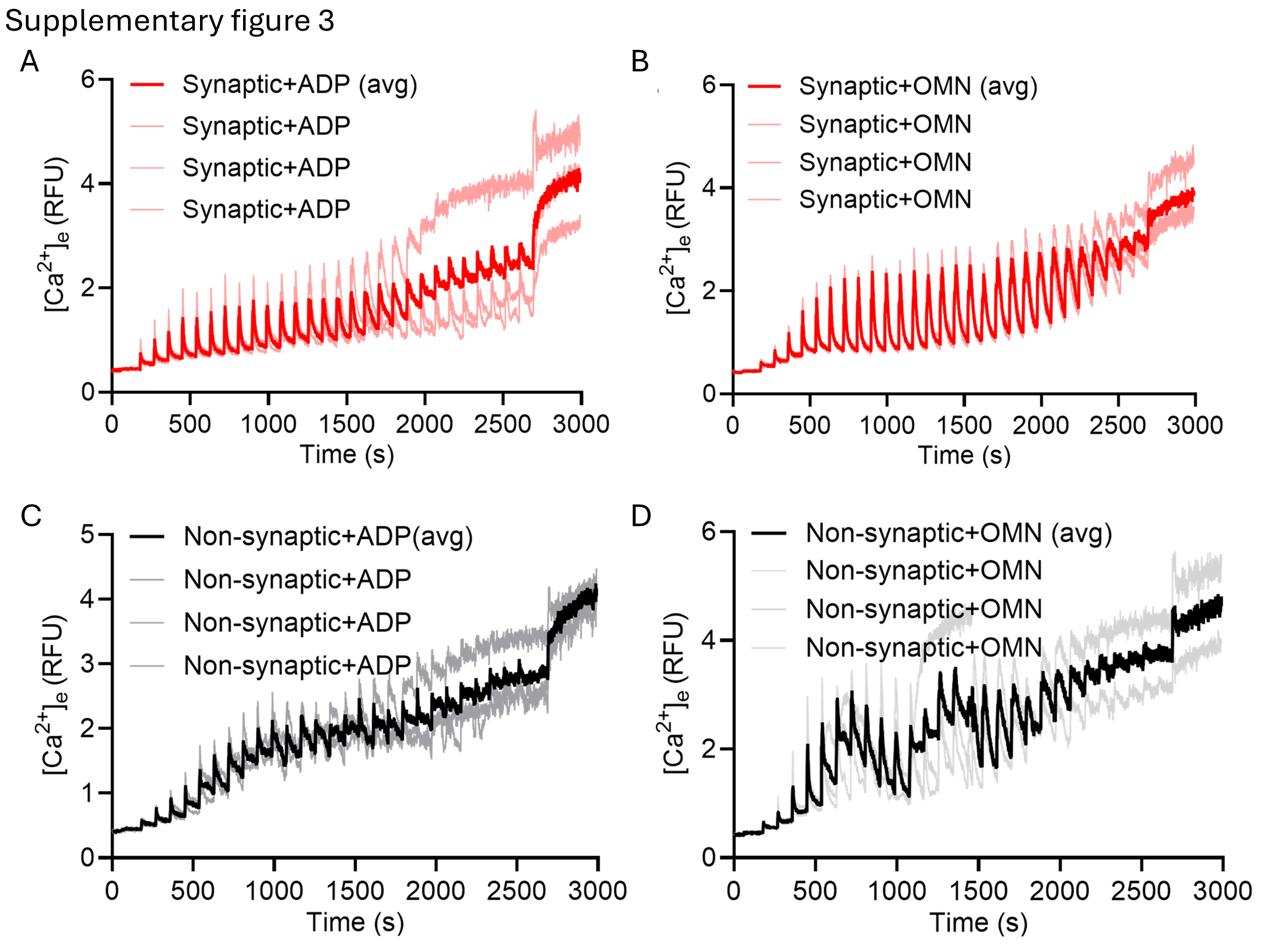

Supplement: SUPPLEMENTARY FIGURE 3 — Effect of ADP alone (A,C) and OMN alone (B,D) on extra-mitochondrial calcium ([Ca2+]e) dynamics of synaptic (A,B) and non-synaptic (C,D) mitochondria. Representative traces of extra-matrix Ca2+ ([Ca2+]e) measured with the Ca2+-sensitive ratiometric dye Fura-4F in isolated synaptic (A,B) and non-synaptic (C,D) mitochondria. 250 μM ADP (A,C) and 10 μM OMN (B,D) were added to synaptic (pink traces) and non-synaptic (gray traces) mitochondria at 30 s followed by the addition of the complex I substrates, [Na+-glutamate + Na+-malate (GM)] at 60 s. 40 μM CaCl2 pulses were added at every 90 s, and 10 μM CCCP was added at the end of each experiment. [file Image_3.TIF]
